# Genetically Determined Serum Calcium Levels and Markers of Ventricular Repolarization: A Mendelian Randomization Study in the UK Biobank

**DOI:** 10.1161/CIRCGEN.120.003231

**Published:** 2021-04-22

**Authors:** William J. Young, Helen R. Warren, Dennis O. Mook-Kanamori, Julia Ramírez, Stefan van Duijvenboden, Michele Orini, Andrew Tinker, Diana van Heemst, Pier D. Lambiase, J. Wouter Jukema, Patricia B. Munroe, Raymond Noordam

**Affiliations:** 1 Clinical Pharmacology Department, William Harvey Research Institute (W.J.Y., H.R.W., J.R., S.v.D., A.T., P.B.M.), Barts and the London School of Medicine and Dentistry, Queen Mary University of London.; 2 NIHR Barts Cardiovascular Biomedical Research Unit (H.R.W., A.T., P.B.M.), Barts and the London School of Medicine and Dentistry, Queen Mary University of London.; 3 Barts Heart Centre, St Bartholomew’s Hospital, Barts Health NHS trust (W.J.Y., M.O., P.D.L.).; 4 Department of Clinical Epidemiology (D.O.M.-K.), Leiden University Medical Center, the Netherlands.; 5 Department of Public Health and Primary Care (D.O.M.-K.), Leiden University Medical Center, the Netherlands.; 6 Department of Internal Medicine (D.v.H., R.N.), Leiden University Medical Center, the Netherlands.; 7 Department of Cardiology (J.W.J.), Leiden University Medical Center, the Netherlands.; 8 Institute of Cardiovascular Sciences, University of College London, United Kingdom (J.R., S.v.D., M.O., P.D.L.).; 9 Netherlands Heart Institute, Utrecht, the Netherlands (J.W.J.).

**Keywords:** action potential, calcium, cardiovascular diseases, electrocardiography, electrolyte

## Abstract

**Background:**

ECG markers of ventricular depolarization and repolarization are associated with an increased risk of arrhythmia and sudden cardiac death. Our prior work indicated lower serum calcium concentrations are associated with longer QT and JT intervals in the general population. Here, we investigate whether serum calcium is a causal risk factor for changes in ECG measures using Mendelian randomization (MR).

**Methods:**

Independent lead variants from a newly performed genome-wide association study for serum calcium in >300 000 European-ancestry participants from UK Biobank were used as instrumental variables. Two-sample MR analyses were performed to approximate the causal effect of serum calcium on QT, JT, and QRS intervals using an inverse-weighted method in 76 226 participants not contributing to the serum calcium genome-wide association study. Sensitivity analyses including MR-Egger, weighted-median estimator, and MR pleiotropy residual sum and outlier were performed to test for the presence of horizontal pleiotropy.

**Results:**

Two hundred five independent lead calcium-associated variants were used as instrumental variables for MR. A decrease of 0.1 mmol/L serum calcium was associated with longer QT (3.01 ms [95% CI, 2.03 to 3.99]) and JT (2.89 ms [1.91 to 3.87]) intervals. A weak association was observed for QRS duration (secondary analyses only). Results were concordant in all sensitivity analyses.

**Conclusions:**

These analyses support a causal effect of serum calcium levels on ventricular repolarization, in a middle-aged population of European-ancestry where serum calcium concentrations are likely stable and chronic. Modulation of calcium concentration may, therefore, directly influence cardiovascular disease risk.

## Abstract 2

Supplemental Digital Content is available in the text.

Noninvasive markers of cardiac disease derived from the ECG are associated with major cardiovascular events and reflect underlying abnormalities in cardiac structure and electrical conduction.^1–4^ Abnormal action potential duration and amplification of the spatial dispersion of repolarization, coupled with early after depolarizations inducing triggered activity is an important mechanism of ventricular arrhythmia, specifically torsades de pointes tachycardia.^5,6^ Prolongation of the QT interval, a marker of the time needed for ventricular repolarization and depolarization, has consistently been associated with adverse outcomes, including ventricular arrhythmia and sudden cardiac death.^7–9^ QRS duration (time point from QRS onset to offset) is specific for ventricular depolarization while the JT interval is specific for ventricular repolarization spanning the interval from QRS offset to T-wave end. Multiple factors may influence these ECG markers and thus the potential for arrhythmia, including mutations in genes encoding ion channels and their accessory proteins (eg, *KCNQ1* and *KCNE1*) and iatrogenic causes due to off target effects by medication (eg, cancer therapeutics and psychotropics).^10–12^

The different phases of the cardiac action potential are caused by the (inward and outward) movement of different ions across the membrane of the cardiac cells. Serum electrolyte concentrations are associated with alterations in ECG-derived indices of cardiac electrophysiological activity. Historically, studies have focused on the effects of electrolytes in clinical populations often with serum electrolyte concentrations significantly outside of the normal range with rapid and acute changes in their concentration.^13,14^ We recently published the results of a large meta-analysis of cross-sectional data including 153 014 unselected individuals, investigating the association of serum electrolyte levels with ECG-derived indices.^15^ One of the key findings was an association between lower serum calcium and longer QT (2.23 ms per 0.1 mmol/L) and JT (2.27 ms per 0.1 mmol/L) intervals but not with QRS duration. The lack of a calcium-QRS duration association suggested serum calcium specifically affects ventricular repolarization. However, given the observational and cross-sectional nature of the study, and the limited number of considered confounders, we were unable to determine whether these observations were causal.

Mendelian randomization (MR), in which genetic variants significantly associated with an exposure are used to estimate causal effects of that exposure on outcomes of interest,^16–18^ has been widely used to assess causality in observational settings. MR overcomes the main limitations of observational studies, notably reverse causation and residual confounding.^19^ Previous genome-wide association studies (GWAS) for serum calcium have identified associated variants and have been leveraged before in MR studies for cardiovascular disease risk.^20–23^ However, due to the relatively small sample sizes of these GWAS which limited the number of associations identified, the genetic instruments included in MR analyses explained only a small proportion of the variance of calcium (≈0.9%).^24,25^ The release of biochemical data in UK Biobank (UKB) permits the identification of additional genetic variants for serum calcium in larger samples increasing the number of variants and consequently increasing the power of an MR study.^26,27^ In this study, we performed a new GWAS on serum total calcium and used the independent lead variants as instrumental variables to assess potential causality of the association between lower serum calcium and prolongation of QT and JT intervals in UKB, including QRS duration as a negative control.

## Methods

**Figure 1.**
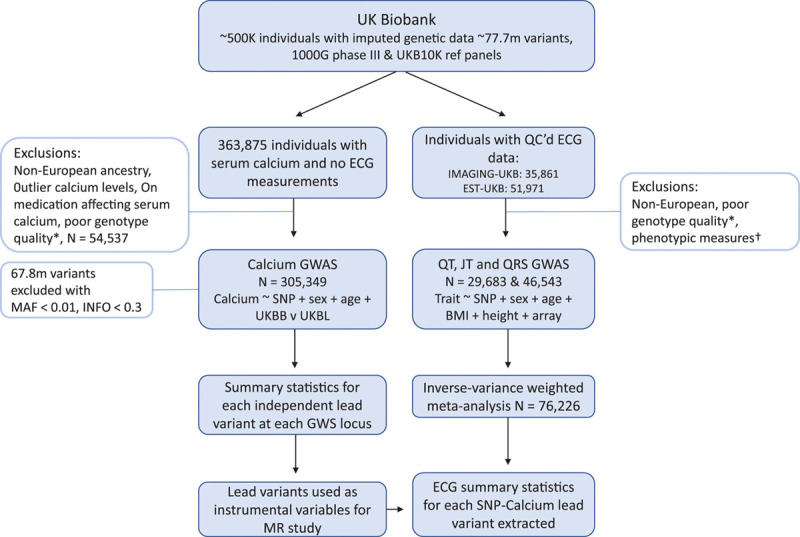
**Workflow indicating the methods for GWAS of serum calcium and ECG traits.** *Phenotypic exclusions included a prior diagnosis of myocardial infarction or heart failure, QRS duration >120 ms or right/left bundle branch block on ECG, pacemaker in-situ, currently pregnant, or taking digitalis medication, class I/III antiarrhythmics or specific QT prolongation medication. †Indicators of poor genotype quality included high heterozygosity/missingness/sex mismatch. Array: indicator for UK Biobank (UKBB) or UK BiLEVE (UKBL) array to adjust for genotyping chip. GWAS indicates genome-wide association study; GWS, genome-wide significant (*P*<5×10^−8^); INFO, imputation quality score; MAF, minor allele frequency; MR, Mendelian randomization; and SNP, single nucleotide polymorphism.

Anonymized clinical, genotype, and ECG data were obtained from UKB.^27^ The UKB study has approval from the National Health Service North West Multi-Centre Research Ethics Committee (ref 11/NW/0382) and participating studies provided informed consent. Any data generated by this study will be returned to UKB in accordance with researcher obligations, to be made available for further research. Full methods are available in Methods in the Data Supplement and also summarized in Figure 1.

## Results

### Calcium GWAS

We identified 208 independent lead variants (201 from novel loci) associated with serum total calcium concentration at genome-wide significance level (*P*<5×10^−8^; Table I in the Data Supplement). A Manhattan plot and quantile-quantile plot are shown in Figures I and II in the Data Supplement. The percentage variance of total serum calcium explained by variants included in this MR study was 5.8% (compared with 0.9% for previously reported variants).^24^ Previously reported variants associated with serum calcium showed the same direction of effect and similar effect size estimates (Table II in the Data Supplement). There were 208 independent lead genome-wide significant variants identified in the albumin-corrected calcium GWAS, of which 151 were in loci overlapping with those reported in the uncorrected calcium GWAS at *P*<5×10^−8^ (Figure III in the Data Supplement). The correlation between results of genome-wide significant loci between the original versus the albumin-corrected GWAS was *r*^2^=0.88 for the β estimates and *r*^2^=0.55 for the *P* values (Spearman rank coefficient). Following exclusion of palindromic single-nucleotide polymorphisms with intermediate allele frequencies, 205 and 202 variants for total serum calcium and albumin-corrected calcium respectively, were included in MR analyses.

## Mendelian Randomization Analyses

### Primary Analysis—Inverse-Variance Weighted

Study characteristics for individuals included in each ECG cohort-specific GWAS and subsequently combined in the meta-analysis, and the calcium GWAS are shown in Table 1. A total of 76 266 participants were included with a median age of 61 (interquartile range: 54–66) years and 53.1% were women.

**Table 1.**
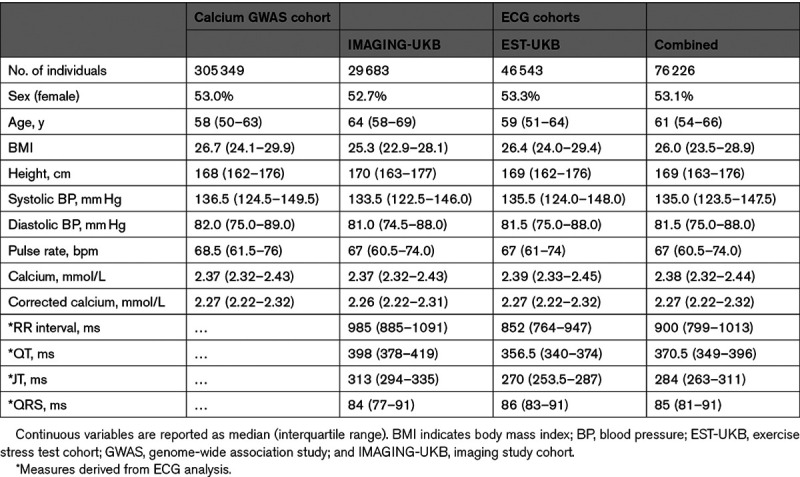
Study Characteristics for Each ECG Cohort and Combined

The results for the estimated causal effect of total serum calcium on the ECG measures are shown in Table 2. Using the inverse-variance weighted model, a genetically determined 0.1 mmol/L decrease in serum total calcium was associated with a 3.01 ms (95% CI, 2.03 to 3.99) longer QT interval and a 2.89 ms (1.91 to 3.87) longer JT interval. No association was found with QRS duration (0.20 ms [−0.10 to 0.49]). The results for albumin-corrected calcium were similar showing the strongest association with QT and JT intervals, but a weak association with QRS duration was observed (0.39 ms [0.08 to 0.69] Table III in the Data Supplement).

**Table 2.**
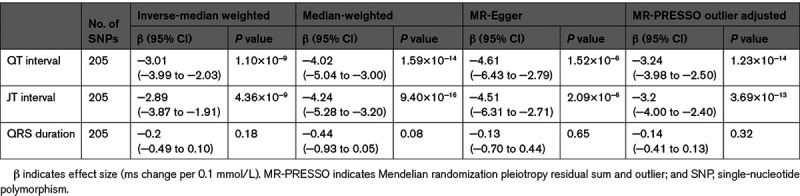
Association Between Serum Total Calcium Concentration and Measures of Ventricular Depolarization and Repolarization Using Mendelian Randomization

### Sensitivity Analyses

Genetically determined lower serum calcium concentrations were consistently associated with longer QT and JT intervals across sensitivity analyses using weighted-median estimator, MR-Egger and Mendelian randomization pleiotropy residual sum and outlier methods, with similar or stronger effect sizes as using the inverse-variance weighted model (Table 2 and Table III in the Data Supplement). Furthermore, we did not observe that any of the intercepts with MR-Egger deviated significantly from zero (*P*>0.05), indicating no evidence of bias from pleiotropy. The results were similar after exclusion of instrumental variants using a more stringent *r*^2^ threshold (>0.001), (Table IV in the Data Supplement).

**Figure 2.**
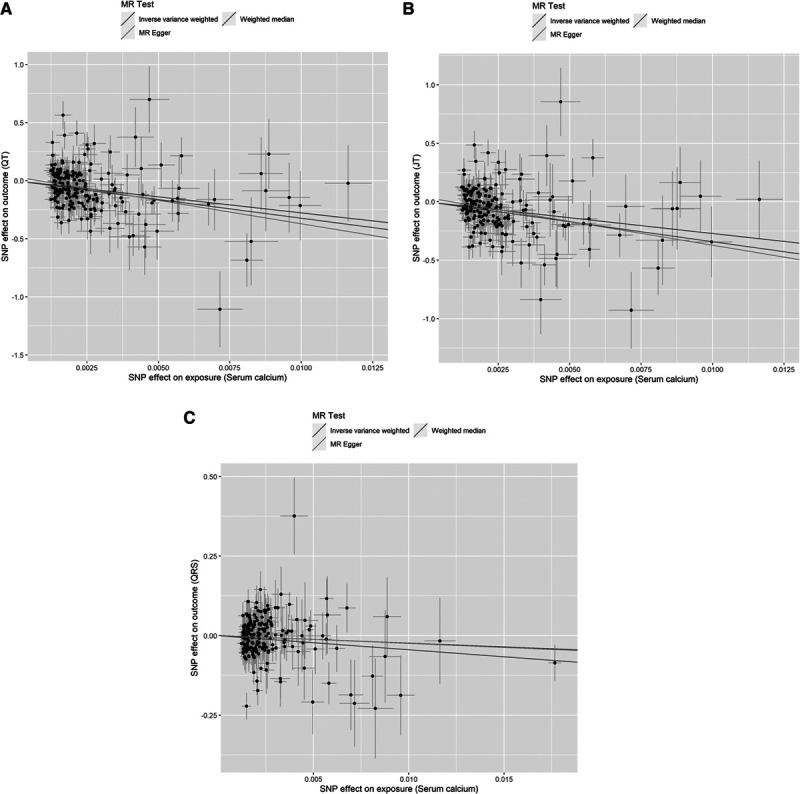
**Scatter plot for Mendelian randomization (MR) serum total calcium analyses for QT, JT and QRS.** Scatter plots of individual variant regression coefficients with inverse-variance weighted, weighted-median, and MR-Egger slope estimates. A, QT. B, JT. C, QRS. SNP indicates single nucleotide polymorphism.

Similar results were identified after exclusion of the variant mapped to *CASR*, a locus, which is a major genetic determinant of serum calcium concentration.^21^ Scatter plots for serum total calcium analyses are presented in Figure 2A through 2C for each ECG measure. Funnel plots did not indicate any directional horizontal pleiotropy (Figure IVA through IVC in the Data Supplement).

## Discussion

This study uses MR to demonstrate the causal inverse relationship between serum calcium concentration and longer QT and JT intervals in UKB, a large middle-aged European ancestry population. This inverse relationship was consistent across all sensitivity analyses. These results along with the absence of a clinically relevant association with QRS duration due to its very small effect size, collectively suggest that a genetically predicted lower serum calcium is a causal contributor primarily for increasing ventricular repolarization time in a population where serum calcium concentration exposure is likely stable and chronic. They also highlight the utility of MR in the investigation of clinically relevant variables and their contribution, to specific time points in ventricular cardiac electrophysiology.

It is well recognized that extremes of both hypocalcemia and hypercalcemia in clinical cohorts result in prolongation and shortening of ventricular repolarization, respectively.^28^ However, there has previously been limited study of the influence of stable calcium concentrations in population-based studies. We previously reported an inverse association between serum total calcium concentration and QT and JT intervals in a large meta-analysis of observational studies with over 150 K unselected individuals.^15^ Specifically, we observed a 2.23 ms longer QT interval and 2.27 ms longer JT interval per 0.1 mmol/L decrease in serum calcium, in the absence of a limited number of considered confounding factors.^15^ These effect size estimates are similar to those obtained in this MR study, using individuals from UKB. It should be noted that UKB was not included in our previous observational meta-analysis study and is thus an independent cohort. When comparing the MR results of serum uncorrected calcium with the secondary analysis using albumin-corrected calcium, our findings were very similar with strong associations identified with QT and JT intervals. The marginal association between serum albumin-corrected calcium and QRS duration was considered not clinically relevant and anticipated given some overlap exists between the genetic contributions of QRS and QT/JT intervals.^29^

Previous randomized control and crossover trials estimated an increase in serum total calcium of 0.07 to 0.13 mmol/L ≈4 hours after ingestion of calcium carbonate (500 mg).^30,31^ Thus, the results of this study suggest oral calcium supplementation could temporarily decrease the QT interval by 2.11 to 3.91 ms. As the effect of oral calcium supplementation on serum total calcium concentration is small, we would expect no direct clinical benefits. However, the results of this study suggest further research into the effects of serum calcium concentration on arrhythmogenesis is warranted and calcium variants could be considered for inclusion in genetic risk score models for risk prediction. This may be of particular importance in patient sub-groups such as endocrinology disorders affecting calcium homeostasis, concurrent use of medication which prolong the QT interval, and in the context of other comorbidities where a substrate exists for ventricular arrhythmia such as ischemic heart disease, cardiomyopathies, or channelopathies.^12,32^

Although an inverse relationship between calcium and markers of ventricular repolarization were identified in this study, associations between higher serum calcium concentrations and increased cardiovascular disease risk including myocardial infarction, stroke, and cardiovascular mortality have been reported in individual epidemiological studies, meta-analyses, and some randomized control trials.^33–35^ These observations are present at serum calcium concentrations within the normal reference range (association at high-normal concentrations). Thus, there is interest in the use of serum calcium levels in the assessment of cardiovascular risk. To date, 6 MR studies have been performed evaluating the effect of calcium on cardiovascular outcomes using 7 independent variants identified from a previous serum calcium meta-analysis (N≈61 000).^24^ Despite the small percentage variance of calcium explained by these variants (≈0.9%), a significant association was identified between serum calcium and coronary artery disease and myocardial infarction, a finding recently replicated in a Mendelian randomization - phenome-wide association study performed in UKB (odds ratio, 1.99 for myocardial infarction per 0.25 mmol/L increase in genetically predicted serum calcium [CI, 1.17–3.39]).^20,36,37^ For atrial fibrillation, an MR study identified no significant association in the main analyses.^21^ However, directional pleiotropy was identified and in MR-Egger analyses, an association was observed (odds ratio, 1.30 per 0.25 mmol/L increase [CI, 1.05–1.59]) driven by a single variant in the *CASR* locus. This variant out of those included as instrumental variables, explained 0.5% of the variance of serum calcium. Significant associations have not been observed with heart failure (as an end point after myocardial infarction) or stroke risk.^22,23^ Additionally, despite calcium supplementation being common in the general population with the intention to reduce the risk of fractures, an association between life-long calcium levels and risk of fracture was not observed in a previous MR study.^38^ However, these studies may have been limited by the low variation of calcium explained by variants included in the MR analyses, despite having large sample sizes for testing these clinical outcomes.^39^

Despite showing evidence for a causal association between lower serum calcium and longer QT and JT intervals, this study does not provide information on the biological mechanisms involved, which remain uncertain. In animal models, the duration of phase II of the cardiac action potential is determined by the inactivation of voltage-gated long-lasting calcium channels, which are dependent on calcium entering these channels and their release from the sarcoplasmic reticulum.^28,40^ Higher extracellular calcium concentrations increase long-lasting calcium channel inactivation which in turn reduces phase II of the action potential and the inverse is present in lower calcium concentration states, as identified in a more recent in-silico theoretical study using a human ventricular myocyte model.^41^ These mechanisms could explain the associations observed in our study between serum calcium and ventricular repolarization.

### Strengths and Limitations

The present study performed a new serum calcium GWAS to increase the number of genetic instrumental variables and to increase the variance explained to perform a more statistically powerful MR analysis. Furthermore, 2-sample MR studies assume the 2 samples (exposure and outcome) were performed in different individuals from the same source population. By design, we performed the new calcium GWAS in individuals not contributing to the QT/JT/QRS intervals GWASs ensuring this assumption was met.

UKB is a densely phenotyped cohort, and participants are generally healthy compared with the general UK population. Additionally, this study was conducted only in individuals of European ancestry due to a limited sample size available for other ancestries. Therefore, these results may not be extrapolated to population groups of non-European ancestry or within high-risk clinical cohorts such as postmyocardial infarction or channelopathies showing a mendelian pattern of inheritance.

### Conclusions

In summary, this MR study indicates that genetically determined lower serum calcium concentrations are causally associated with longer ventricular repolarization time in a middle-aged population where serum calcium concentration exposure is likely stable and chronic. Modulation of calcium concentration may, therefore, directly influence cardiovascular disease risk. Additionally, we have shown that the power of MR studies can be harnessed to improve our understanding of cardiac electrophysiology, and a similar approach could be considered using other clinically relevant exposures.

## Acknowledgments

This research has been conducted using the UK Biobank Resource (application 8256—Understanding genetic influences in the response of the cardiac electrical system to exercise).

## Sources of Funding

Dr W.J. Young is supported by a Medical Research Council (MRC) grant MR/R017468/1. This research has been conducted using the UK Biobank Resource (application 8256—Understanding genetic influences in the response of the cardiac electrical system to exercise) and is supported by MRC grant MR/N025083/1. Dr W.J. Young, Dr Warren, Dr Ramírez, Professor Tinker, Professor Lambiase, and Professor Munroe acknowledge the National Institute for Health Research (NIHR) Cardiovascular Biomedical Centre at Barts and The London, Queen Mary University of London. Professor Lambiase is supported by University College London/University College London Hospitals NHS Foundation trust UCL/UCLH Biomedicine NIHR, Barts Heart Centre Biomedical Research Centre. Dr Ramírez acknowledges support from the European Union’s Horizon 2020 research and innovation programme under the Marie Sklodowska-Curie grant agreement No.786833.

## Disclosures

Dr Mook-Kanamori is a part time research consultant at Metabolon, Inc. The other authors report no conflicts.

## Supplemental Materials

Supplemental Methods

Supplemental Tables I–IV

Supplemental Figures I–IV

References ^51–57^

## Supplementary Material


