# Supplementary material for: Genetically Determined Serum Calcium Levels and Markers of Ventricular Repolarization: A Mendelian Randomization Study in the UK Biobank
Source: Circ Genom Precis Med. 2021 Apr 22;14(3):e003231. doi: 10.1161/CIRCGEN.120.003231 (PMC8208093; doi:10.1161/CIRCGEN.120.003231)
Supplement: Supplementary file 1 [file hcg-14-e003231-s001.pdf]

SUPPLEMENTAL MATERIAL

Supplementary Methods

I Study cohort information

II Calcium GWAS for the selection of the genetic instrumental variables

II.I Biochemistry data – calcium collection

II.II Selection of individuals for inclusion in serum Calcium GWAS

II.III Serum Calcium Genome-wide association analysis

III QT, JT and QRS interval GWAS for summary statistics to use as outcomes in mendelian randomisation analyses

III.I QT, JT and QRS interval measurement

III.II Study population for QT, JT and QRS intervals GWAS

III.III Genome wide association analysis for QT, JT and QRS intervals

IV Calculation of locus boundaries

V Mendelian Randomisation study

Supplementary Tables I-IV

Supplementary Figures and Figure Legends I-IV

Supplementary Methods

I Study Cohort information

The UKB is a prospective study of approximately 500,000 individuals aged 40-69 years at recruitment (2006-2008) with genetic data and a rich source of phenotypic traits including baseline clinical measurements and serum biochemistry data[27]. The UKB study has approval from the North West Multi-Centre Research Ethics Committee, and all participants provided informed consent. As part of an imaging study (IMAGING-UKB), 12-lead resting ECGs were available for 36,504 individuals recorded between May 2014 – September 2019. Additionally, 79,772 individuals participated in a stationary bicycle exercise test (EST-UKB) with 1-lead ECG data (Cardiosoft v6.51) from a 15 second resting phase before activity. Genetic data was available for 488,377 individuals. Genotyping was performed using the Affymetrix UK BiLEVE Axiom array on an initial 50,000 participants. The remaining 450,000 participants were genotyped using the Affymetrix UK Biobank Axiom® array that genotyped ~850,000 variants. Single nucleotide polymorphisms (SNPs) were then imputed using a merged UK10K haplotype reference panel and 1000 Genomes Phase 3 reference panel using IMPUTE2 software.

II Calcium GWAS for the selection of the genetic instrumental variables

II.I Biochemistry data – calcium collection

Blood samples were obtained during the baseline visit. The average time from venepuncture to storage was 24 hours (+/- 2.5hrs). Calcium was measured using a Beckman Coulter (UK) Ltd assay and Beckman Coulter AUS800 platform using colourmetric analysis methodology. Units of measurement were mmol/L and the manufacturers analytical range was 1-

5 (mmol/L). Extensive QC procedures were followed to identify invalid results, dilution issues and laboratory drift - the details of this can be identified on the UKB website[26].

II.II Selection of individuals for inclusion in serum Calcium GWAS

363,875 UKB participants had serum calcium levels with no ECG measurements (these were excluded to have no overlap in individuals between the analyses). Participants were clustered into ethnic groups according to genotype principal components 1 and 2, using the K means function in R version 3.5.3. After clustering, 337,011 individuals had genotype data and were of European ancestry. Individuals of poor genotype quality (high heterozygosity or missingness) and sex-discordance were excluded (N = 1,166). Individuals with outlier calcium measurements (> +/- 5SD of mean) were excluded (N=249). Participants taking medication known to influence serum calcium levels including calcium supplementation, calcitonin, bisphosphonates and thiazide diuretics were excluded (n = 30,247). The final sample size for the Calcium GWAS was 305,349.

II.III Serum Calcium Genome-wide association analysis

Variants were excluded from analysis if minor allele frequency (MAF) < 0.01 or imputation quality score < 0.3. Association analyses were performed using a linear mixed model with BOLT-linear mixed model (LMM) software, which uses LD structure to take in to account population structure and relatedness[42]. Covariates for age, sex and genotyping array (either UK Biobank or UK BiLEVE array) were included in the model. In addition to a serum total calcium GWAS, a secondary analysis was performed using calcium corrected for serum albumin concentration, calculated using the formula: Albumin-corrected calcium = Total serum calcium (mmol/L) + (0.02 * (40 – Albumin (g/L))[43,44]. Quality control of GWAS summary statistics was

performed including manual inspection of Quantile- Quantile (QQ), *P*-value and Z-statistics (P-Z) and allele frequency (AF) plots using the EasyQC package in R[45]. Genome-wide significance threshold was set at $P < 5\times10^{-8}$. The LD score regression intercept was calculated using LDSC, to evaluate for polygenicity[46].

III QT, JT and QRS interval GWAS for summary statistics to use as outcome in mendelian randomisation analyses

III.I QT, JT and QRS interval measurement

Of the EST-UKB sub-cohort, only ECG signals from the initial resting phase (15 seconds) prior commencing exercise were used. 12-lead resting ECGs from the IMAGING-UKB cohort were analysed separately. Lead I was used for calculating QT, JT and QRS intervals for consistency across cohorts. ECG signal processing and fiducial point identification was performed using Matlab version 2018b[47]. A bandpass Butterworth filter was applied ([0.5, 45]) for noise reduction while limiting the impact on the QRS complex morphology. ECGs with fewer than 5 QRS complexes or an intra-beat correlation of less than 0.8 were excluded. Using the first principal component, R peak was identified by taking double the difference of the signal and applying a threshold of 1.5x the standard deviation of the resulting signal. Signal averaging was implemented to further reduce noise as in previous studies[48]. Signal-averaged ECGs were produced using only beats with a similar morphology (correlation coefficient > 0.8). The Hilbert's envelope method was used to identify QRS onset and offset[49]. This creates an 'envelope' of the QRS complex by taking the magnitude of the signal within a pre-defined window. Marker location was obtained from the envelope by taking the tangent from the first derivative before and after the R peak, to the isoelectric baseline. T wave end was calculated as the tangent from the minimum of the first

derivative of the T wave slope, to the isoelectric line. QT was the time interval from QRS onset to T end, QRS duration was the time interval from QRS onset to QRS offset and JT interval was calculated as QT – QRS. All intervals, including RR interval, were measured in milliseconds (ms). Outliers were manually reviewed (approximately 5% of ECGs) using a locally produced ad-hoc Graphical User Interface in Matlab. In total, 35,861 and 51,971 ECGs passed quality control for IMAGING-UKB and EST-UKB sub-cohorts respectively.

III.II Study population for QT, JT and QRS intervals GWAS

If individuals had ECG measurements from both tests, only the result from one test was included, with the 12-lead resting ECG result preferred. Participants were excluded if QRS duration was greater than 120ms, right or left bundle branch block on ECG, a prior history of myocardial infarction, heart failure, pacemaker of implantable cardiac defibrillator implantation, or if pregnant at the time of ECG acquisition. Additionally, participants using digitalis medication, class I or III anti-arrhythmics or specific QT prolongation medication were also excluded (N = 6,201 and 5,429 for IMAGING-UKB and EST-UKB respectively). In total, 29,683 and 46,543 individuals were included from IMAGING-UKB and EST-UKB cohort respectively.

III.III Genome wide association analysis for QT, JT and QRS intervals

The method for GWAS was the same as per serum calcium GWAS (section 2.3) except for the following differences. A GWAS was performed separately for IMAGING-UKB cohort and EST-UKB cohorts and then meta-analysed, to take into account, the different methods of obtaining the ECG recording. Covariates used for GWAS were age, sex, RR interval, BMI, height and genotyping array. As also done in other GWAS studies for these traits, BMI and height were both

included as BMI alone is an inaccurate estimate of an individual's geometric dimensions[29,50,51]. Both were significant in a multi-variable linear regression models with these ECG traits. The spearman correlation coefficients between BMI and height were only 0.05 and 0.06 for IMAGING-UKB and EST-UKB cohorts respectively. Following quality control of the two separate GWAS analysis results, an inverse variance weighted fixed effects model meta-analysis was performed using METAL (version released 2011-03-25)[52]. Genomic control was applied to each sub-study if lambda was > 1.

IV Calculation of locus boundaries

Pairwise LD was calculated using UKB participants included in the calcium GWAS using PLINK v1.9[53]. Variants were ordered according to chromosome and position and LD calculated in a 4mb region centred on each lead variant. Variants with an $r^2 < 0.1$ from the lead variant (smallest P-value) were removed. The start position was defined as the position of the first variant in this order minus 50kb and the end position defined as the last variant in the order plus an additional 50kb. Overlapping windows were merged to produce the final genome start and end position for the locus. This window was declared the genomic window for the locus or a window of +/- 500kb around the lead variant, which ever was larger..

V Mendelian Randomisation study

Summary-level data for each genome-wide ($P < 5x10^{-8}$) significant single-nucleotide polymorphism (SNP)-Calcium lead variant at each locus (defined as a region $r^2 < 0.1$ or ± 500kb centred on the lead variant, whichever was larger), was extracted from each meta-analysis result for QT, JT and QRS (**Supplementary Table 1, Data Supplement**). The MRC Integrative

Epidemiology Unit ((MRCIEU) TwoSampleMR package in R was used to harmonise the direction of effects between exposure and outcome SNPs and to perform the MR analyses[18]. Palindromic variants were excluded if they had intermediate allele frequencies preventing inference of positive strand alleles. For our main analyses, we used the inverse variance-weighted (IVW) approach[54], which generates the estimated causal estimate by regressing the SNP-ECG measure estimate on the SNP-Calcium association weighted for the inverse of the SNP-ECG standard error. To facilitate the clinical interpretation of the study results, our MR results are presented as the difference in QT, JT and QRS interval in ms per 0.1 mmol/L lower serum calcium. Our primary MR analysis was performed using variants from the uncorrected serum total calcium GWAS. We also performed a secondary analysis using lead variants from the albumin-corrected calcium GWAS.

As the presence of pleiotropic variants is likely with large numbers of variants used as genetic instrumental variables, we explored whether such variants influence the causal estimate (directional pleiotropy) by performing sensitivity analyses using MR-Egger[55], weighted-median estimator[56] and MR pleiotropy residual sum and outlier (MR-PRESSO)[57] methods. MR-Egger is analogous to IVW but does not force the regression line through an intercept of zero. It is statistically less efficient (providing larger standard errors) but provides a causal estimate that is corrected for potential directional horizontal pleiotropy under the InSIDE (INstrument Strength Independent of Direct Effect) assumption that the direct pleiotropic effects of genetic variants on the outcome are distributed independently from the genetic associations with the exposure[55]. A significant deviation from the intercept is considered to be evidence for presence of directional pleiotropy in the analyses[55]. The weighted-median estimator calculates normalized inverse-variance weights for each genetic variant and decreases the contribution of variants with outlying

ratio estimates[56]. It is valid if more that 50% of the weight of the genetic instrument is from valid variants (i.e if one SNP or several SNPs jointly contribute 50% or more of the weight and exhibit horizontal pleiotropy, the effect estimate may be biased)[56]. MR-PRESSO detects outlier genetic instrumental variables and is best suited when horizontal pleiotropy occurs in <50% of the instruments[57]. To avoid over-precise estimates due to (moderately) correlated variants, we additionally performed a sensitivity analysis excluding genetic instrumental variables with an $r^2 > 0.001$.

Supplementary Table I: Lead variants at each genome wide significant locus for serum total calcium

SNP	Chr	Position	Effect Allele	EAF	Beta	SE	*P*-value	INFO score
rs61766352	1	1086179	C	0.2787	-0.002376	0.0002565	2.80E-22	0.963922
rs36086195	1	16510894	T	0.5791	0.001839	0.0002291	1.10E-15	0.996606
rs12132412	1	21820042	G	0.3906	0.002386	0.0002337	1.70E-20	0.978882
rs1184556173	1	23747996	G	0.7264	0.001864	0.0002571	1.50E-14	0.973588
rs111745074	1	39792369	T	0.2105	-0.001546	0.0002776	1.20E-08	0.999225
rs841572	1	43436051	A	0.4069	0.002805	0.0002307	1.60E-35	0.993747
rs112174050	1	51581471	T	0.02497	0.009962	0.0007328	1.70E-43	0.972946
rs148756772	1	68270398	C	0.03229	0.006967	0.0006552	4.10E-27	0.953719
rs1848837	1	78578550	G	0.2881	-0.001482	0.0002502	3.70E-10	0.995229
1:91544957_ACT_A	1	91544957	A	0.1389	-0.002026	0.0003485	2.40E-08	0.886017
rs629301	1	109818306	T	0.778	0.00158	0.000272	9.40E-10	1
1:112218092_TAAAAAAAACAAAAC_T	1	112218092	T	0.1624	0.001677	0.0003076	1.00E-08	0.995384
rs10737767	1	116830250	C	0.4161	0.001402	0.0002306	6.50E-10	0.987834
1:150878099_TC_T	1	150878099	T	0.2202	0.002145	0.000273	6.50E-16	0.998053
rs760077	1	155178782	T	0.6011	-0.002513	0.0002307	1.50E-26	1
1:178502207_GTA_G	1	178502207	G	0.3434	-0.001531	0.0002389	4.80E-11	0.995427
rs1434282	1	199010721	T	0.725	0.002059	0.0002543	9.20E-17	0.989815
rs4506486	1	200453320	T	0.6019	0.001539	0.0002312	3.50E-11	0.996444
rs1497826	1	217471419	G	0.3736	0.001792	0.0002351	2.80E-15	0.984554
rs3011	1	220085453	C	0.3689	-0.001904	0.0002353	2.70E-16	0.989628
rs6667260	1	226923938	C	0.4579	-0.0016	0.0002269	1.00E-13	1
2:9032564_CCATT_C	2	9032564	C	0.02193	0.007157	0.000789	5.30E-20	0.952076
rs34636567	2	25508812	TA	0.5224	-0.001481	0.0002302	2.50E-10	0.960841
rs1260326	2	27730940	C	0.6051	-0.004453	0.0002309	4.30E-87	1
rs12467820	2	37572806	T	0.4936	0.001244	0.0002266	2.70E-09	0.997426
rs10193807	2	39601264	C	0.8402	0.001816	0.0003098	5.80E-09	0.990732
rs6545412	2	54727795	T	0.1538	0.002016	0.0003164	1.50E-10	0.979506

rs148785604	2	61366095	TTTCCTTCC	0.5615	-0.002601	0.0002282	5.90E-31	0.993168
rs12468920	2	66197123	G	0.307	0.001388	0.0002464	2.20E-08	0.985764
rs10647217	2	85453713	TCTC	0.6138	0.001232	0.0002359	2.30E-08	0.967938
rs547408441	2	89864905	A	0.04357	-0.004401	0.0006791	9.30E-12	0.663365
rs111712978	2	91928755	A	0.04736	-0.004198	0.0006374	1.70E-11	0.691832
rs6576983	2	97454650	G	0.3919	-0.003502	0.0002315	2.30E-53	1
rs1448208	2	112263474	T	0.3358	-0.001389	0.0002415	5.10E-09	0.978167
rs895418	2	114018320	T	0.4693	0.002143	0.0002275	2.40E-21	0.990836
rs11673819	2	121070729	T	0.7035	-0.002192	0.0002485	8.40E-19	0.989342
rs13389219	2	165528876	T	0.3928	-0.00139	0.0002313	1.30E-10	0.999668
rs12613807	2	191343763	C	0.4449	0.001483	0.0002275	4.30E-11	1
rs7559013	2	213735555	C	0.1295	0.002221	0.0003373	8.70E-11	0.994244
rs838717	2	234296444	A	0.5667	-0.003746	0.0002285	1.00E-60	0.995463
rs10197122	2	240285317	T	0.0721	0.002422	0.0004388	1.40E-08	0.989078
rs60624480	2	242297118	A	0.3596	-0.002183	0.0002355	1.90E-21	0.999284
rs35154162	3	4629789	T	0.7695	-0.00191	0.0002681	1.90E-14	0.9941
rs1801282	3	12393125	G	0.1197	-0.00332	0.0003469	2.20E-22	1
rs11709284	3	52559705	A	0.5551	0.001386	0.0002271	3.60E-10	0.999167
rs12485738	3	56865776	G	0.629	-0.001704	0.0002335	3.00E-13	1
rs531500623	3	101306098	AAG	0.3262	-0.001398	0.0002418	9.20E-09	0.987113
rs16853573	3	107233412	C	0.07673	0.004122	0.0004299	7.40E-22	0.967897
rs2271494	3	113300183	T	0.4164	0.001836	0.0002293	8.70E-17	0.996518
rs73186030	3	122013465	C	0.869623	-0.0176658	0.000335171	2.7E-616	0.995978
rs28519617	3	135874930	G	0.2703	0.002542	0.0002555	9.60E-24	0.988605
rs3773912	3	152180137	T	0.463	-0.001284	0.0002267	1.00E-08	0.99302
rs13322435	3	156795468	G	0.4039	0.001779	0.0002313	1.90E-15	0.989521
rs2593813	3	186332571	A	0.6342	0.003279	0.0002344	5.70E-47	0.993853
rs13108218	4	3443931	G	0.6157	-0.003487	0.0002348	3.10E-51	0.98165
rs372867519	4	27001674	D	0.2722	-0.002003	0.0002581	1.60E-15	0.970681
rs7688574	4	38533499	T	0.3707	0.001386	0.0002348	3.10E-09	0.995798
rs6841258	4	40565426	T	0.1678	-0.003692	0.0003026	9.00E-36	1

rs4864709	4	53879085	C	0.3711	0.001293	0.0002347	2.50E-08	1
rs13111128	4	56444321	A	0.3031	-0.001299	0.0002492	6.80E-09	0.976552
4:75945702_TCC_TC	4	75945702	TC	0.06544	-0.002584	0.0004601	2.20E-08	0.982898
rs13107325	4	103188709	T	0.0758	-0.006227	0.0004283	2.50E-48	1
rs2713844	4	106287354	A	0.7632	-0.00165	0.0002668	3.20E-10	0.998065
rs2388993	4	115379445	G	0.5869	-0.001311	0.0002303	3.70E-09	0.998831
rs4320103	4	170910322	G	0.03895	0.00452	0.0005913	2.80E-13	0.972779
rs35841312	5	72235092	GA	0.3189	0.001835	0.0002448	1.50E-15	0.983735
rs10942734	5	74595194	C	0.4284	0.001531	0.0002294	2.20E-11	0.995173
rs6897362	5	76110217	A	0.5261	-0.001326	0.0002268	2.10E-08	0.998696
rs251391	5	127538116	T	0.7453	0.001483	0.0002613	4.50E-09	0.986917
5:131121243_CA_C	5	131121243	C	0.8669	0.002008	0.0003348	1.50E-09	0.990767
rs12519940	5	133904597	T	0.2785	-0.002307	0.0002545	3.60E-20	0.984318
rs6556313	5	176792491	G	0.3318	0.001649	0.0002409	1.30E-12	0.995789
6:26025437_AT_A	6	26025437	A	0.5675	-0.001406	0.0002313	2.40E-10	0.974657
rs71548342	6	28486674	GCA	0.1743	-0.002227	0.0003193	2.70E-12	0.872306
rs7742369	6	34165721	G	0.1748	0.003858	0.0002981	2.00E-37	1
rs1214761	6	43354431	G	0.6787	-0.00133	0.0002427	1.60E-08	0.999518
rs4263551	6	74486371	C	0.5165	-0.003464	0.0002267	1.60E-53	0.9994
rs4946137	6	116418112	A	0.4015	0.001687	0.0002315	3.60E-14	0.998292
rs9388399	6	125333797	C	0.3113	-0.002087	0.0002454	4.10E-18	0.995797
rs7756870	6	130373648	G	0.6886	-0.002046	0.000245	3.30E-18	0.996568
rs1763519	6	134518919	C	0.6065	-0.002531	0.0002322	3.10E-29	0.998017
rs2327774	6	137275839	C	0.3792	-0.001815	0.0002381	2.00E-14	0.958875
rs3857708	7	16140077	A	0.2998	0.001881	0.000249	4.90E-14	0.988455
rs4718271	7	65212402	C	0.4502	0.002856	0.0002279	5.50E-38	0.996128
rs7786368	7	77500734	C	0.4169	-0.0019	0.00023	1.90E-16	0.999052
rs5745687	7	81359051	T	0.06607	0.002584	0.0004565	1.00E-08	1
7:92258733_AAG_A	7	92258733	A	0.2671	-0.002034	0.0002566	4.40E-16	0.99526
rs511220	7	105314015	A	0.4081	0.001341	0.0002424	9.70E-09	0.899524
rs2283038	7	106835410	T	0.2367	-0.002077	0.0002678	7.70E-15	0.992137

rs34372369	7	143092269	A	0.05172	0.003306	0.0005119	4.10E-11	1
7:150502468_AT_A	7	150502468	A	0.1106	0.003132	0.000362	1.20E-18	0.991446
rs4240624	8	9184231	A	0.9094	0.005811	0.0003957	1.00E-49	0.999165
rs62493995	8	21946301	G	0.2876	-0.001932	0.0002593	1.00E-13	0.927084
rs36104352	8	23377604	C	0.1205	0.002472	0.000348	2.40E-12	1
rs9657190	8	38295928	G	0.2464	0.001851	0.0002629	3.90E-12	0.996915
rs111291669	8	63946542	TTA	0.3044	-0.001497	0.0002488	1.20E-08	0.982279
rs74677734	8	72528849	A	0.04885	0.003259	0.0005262	4.60E-10	1
rs6983263	8	98835226	C	0.4744	-0.001321	0.0002288	6.00E-09	0.983778
rs3133528	8	101727388	C	0.181	0.001792	0.0003342	2.90E-08	0.792492
rs6993770	8	106581528	T	0.2871	-0.001879	0.0002503	4.00E-15	1
rs28601761	8	126500031	G	0.419	-0.001306	0.0002324	9.30E-10	0.973857
rs62522556	8	145005450	C	0.3987	0.001577	0.000232	1.90E-11	0.996321
rs4740965	9	903461	A	0.7668	-0.002325	0.0002727	5.30E-18	0.96222
rs77798356	9	4762052	G	0.1217	0.00211	0.0003565	5.10E-10	0.942352
rs11144001	9	71467135	T	0.4715	-0.00216	0.0002281	3.10E-22	0.990852
rs148349564	9	77466700	C	0.0804	-0.003382	0.0004202	8.30E-17	0.98273
rs4744854	9	80498559	C	0.6273	-0.002734	0.0002349	1.90E-32	0.99182
rs12337706	9	97523154	G	0.06085	-0.005103	0.0004742	8.20E-29	0.999036
rs10819178	9	129294976	G	0.6378	0.003035	0.0002375	2.40E-39	0.981918
rs13283282	9	131465481	G	0.146	-0.001994	0.0003211	2.00E-10	1
rs9411378	9	136145425	A	0.222	-0.001617	0.0002843	1.40E-08	0.91984
9:140358581_C_T	9	140358581	T	0.07518	0.002522	0.000438	2.00E-08	0.963691
rs498490	10	8118677	T	0.1661	-0.002636	0.0003052	8.80E-19	0.995632
10:9329035_AT_A	10	9329035	A	0.3259	-0.004779	0.0002475	1.20E-83	0.952875
rs3011642	10	22394843	T	0.2422	0.001886	0.0002649	1.10E-12	0.993735
rs2377965	10	50510522	G	0.5284	-0.001871	0.000227	1.20E-16	0.996384
rs1262217	10	65483262	A	0.8256	0.001964	0.0002984	8.50E-12	1
rs5786388	10	80999929	CA	0.578	0.002082	0.0002315	9.90E-20	0.977379
rs9420589	10	94450233	T	0.4345	0.001641	0.0002289	4.10E-13	0.995257
rs2274224	10	96039597	C	0.4346	-0.001703	0.0002285	2.70E-13	1

rs2296436	10	100179851	C	0.08461	-0.002759	0.000407	8.70E-12	1
rs2419886	10	115841641	T	0.2555	-0.001604	0.000261	1.90E-10	0.989738
rs546082321	10	120980336	CA	0.4009	-0.001373	0.0002487	3.40E-08	0.860137
11:3006027_ACAG_A	11	3006027	A	0.3054	-0.002431	0.0002477	6.60E-24	0.985306
rs7947953	11	10684060	G	0.6123	0.001311	0.0002323	1.20E-08	0.995677
rs11511848	11	13505549	T	0.6261	0.002855	0.0002342	1.70E-33	0.997559
rs373709809	11	33791400	G	0.3355	-0.001404	0.0002418	2.10E-09	0.983497
rs12363232	11	47623890	T	0.3491	-0.001991	0.000238	3.50E-18	1
rs2298615	11	65352062	T	0.2324	-0.00178	0.0002685	4.90E-12	1
rs36225067	11	69453985	C	0.02615	0.005728	0.0007218	9.10E-15	0.959673
rs7115200	11	71752160	G	0.4384	-0.001457	0.0002285	6.50E-11	1
rs302650	11	87901236	A	0.4329	-0.001847	0.0002285	3.40E-16	0.998205
rs561830	11	119043745	T	0.2518	-0.00216	0.0002617	5.10E-16	0.991891
rs11218721	11	122504717	A	0.3894	0.001554	0.0002337	1.30E-12	0.982521
rs7928577	11	126227723	T	0.07379	-0.005488	0.0004333	2.50E-37	0.997659
rs2855799	11	128737694	A	0.8116	-0.001643	0.0002906	1.10E-08	0.994485
rs117213754	12	4006794	A	0.01472	0.00825	0.0009405	3.80E-19	1
rs4763297	12	12889289	C	0.4323	0.001603	0.0002299	6.10E-12	0.989853
rs12369443	12	20582651	G	0.199	-0.001637	0.0002857	3.00E-09	0.989171
rs12320328	12	25408464	G	0.08462	-0.002646	0.0004081	3.40E-11	0.99468
rs79295634	12	47180008	G	0.06783	0.002446	0.0004511	2.70E-08	0.993588
rs199785243	12	49106360	CT	0.3251	-0.002447	0.0002426	1.70E-25	0.995975
rs6580981	12	54723028	A	0.4584	-0.00176	0.0002287	4.50E-14	0.988718
rs11172256	12	57976118	G	0.2558	-0.00192	0.0002603	1.90E-13	0.995783
12:90166745_GTAGA_G	12	90166745	G	0.7739	-0.002422	0.0002764	5.90E-19	0.958548
12:110749537_TA_T	12	110749537	T	0.3492	-0.001488	0.0002458	4.00E-10	0.934599
rs17884869	12	123519112	A	0.02463	-0.009579	0.0007304	8.00E-39	1
rs573849602	13	31035467	GA	0.2634	-0.001557	0.0002619	3.30E-09	0.967043
rs7319447	13	42551508	G	0.1513	-0.003277	0.0003162	2.00E-25	0.99862
rs78844280	13	50349898	A	0.02206	-0.004317	0.0007712	1.70E-08	1
rs12583851	13	110504964	C	0.7503	-0.002021	0.000262	6.40E-15	0.998352

rs8011945	14	21871999	G	0.9093	0.002638	0.0003955	9.00E-11	0.998416
rs11625026	14	24869746	T	0.3309	0.001541	0.000244	1.10E-09	0.972228
rs17718872	14	51000770	T	0.1445	0.001733	0.0003234	1.10E-08	0.997609
rs35852840	14	64595763	A	0.05802	0.002627	0.0004882	6.90E-09	0.986792
rs7144433	14	90850229	T	0.8923	-0.002755	0.0003665	1.20E-14	0.996291
rs17127600	14	92279983	G	0.171	-0.00233	0.0003027	7.80E-14	0.988463
rs28929474	14	94844947	T	0.01967	0.01164	0.0008173	1.70E-46	1
rs75153054	14	105968585	C	0.2474	-0.001828	0.0002706	2.00E-11	0.946817
rs35056049	15	40327793	C	0.7133	-0.001501	0.0002512	4.40E-10	1
rs147233090	15	44028047	T	0.02481	0.008869	0.0007339	1.10E-33	0.985182
rs11632520	15	49358308	T	0.1692	-0.002245	0.0003033	1.00E-13	0.994254
rs12911748	15	51493568	T	0.5221	-0.001535	0.0002291	1.30E-12	0.980632
rs55754498	15	60883394	T	0.05317	-0.003008	0.000507	2.00E-08	0.996853
rs11071896	15	66821250	G	0.25	0.001657	0.0002618	1.00E-09	1
rs34066945	15	69612053	T	0.358	-0.002096	0.0002369	1.40E-19	0.993624
rs12148513	15	75222225	T	0.3792	0.001276	0.0002357	1.70E-08	0.980049
rs11629876	15	96666402	T	0.331	-0.001408	0.000241	3.70E-09	0.999393
rs2745205	16	1844226	G	0.6981	-0.001306	0.0002475	4.10E-08	0.996899
rs41278174	16	16259596	A	0.02739	0.004561	0.0006946	1.00E-10	1
rs16945716	16	47980267	C	0.04904	-0.00359	0.0005266	5.90E-12	0.991843
rs12922549	16	54451747	T	0.2378	-0.00225	0.0002722	6.50E-17	0.959158
rs1858800	16	73024276	T	0.3453	0.002785	0.0002399	4.90E-33	0.984948
rs35610022	16	81586901	T	0.5206	0.001374	0.0002283	1.20E-09	0.98829
rs12918968	16	88520452	C	0.4384	-0.002643	0.0002288	1.90E-32	0.993662
rs34422500	16	89703797	T	0.1304	0.002444	0.0003467	7.10E-14	0.945914
rs11078597	17	1618363	C	0.1864	0.004854	0.0002912	2.50E-64	1
17:6614859_TTTTGTGTGTGTG_T	17	6614859	T	0.1398	0.002457	0.000341	2.00E-13	0.916928
rs12600694	17	18091019	A	0.1998	0.001871	0.0002833	4.10E-11	0.999593
rs66634575	17	27499881	T	0.1831	0.001721	0.0002939	3.60E-09	0.993411
rs36030405	17	37513075	AT	0.2349	0.002508	0.0002737	2.90E-21	0.95251
rs5033	17	42326258	T	0.6999	-0.001606	0.0002495	6.00E-11	0.980931

rs7221118	17	47907641	C		0.2142	-0.001846	0.0002773	3.40E-11	0.992488
rs9910998	17	55292765	A		0.07198	0.003908	0.0004403	2.00E-18	0.992118
rs2270114	17	59478776	C		0.7336	-0.002299	0.0002567	9.20E-20	0.997526
rs77542162	17	67081278	G		0.02267	-0.008596	0.0007621	3.10E-31	1
rs10852764	17	73503489	C		0.9034	0.002144	0.0003836	2.60E-08	0.998444
rs689049	18	57168498	C		0.5811	0.001359	0.0002315	6.80E-09	0.984176
rs55722786	18	60206800	T		0.2847	0.001411	0.0002526	7.70E-09	0.989269
rs190424317	19	3094215	G		0.1368	-0.005693	0.0003685	6.80E-54	0.80207
rs7252372	19	14172896	C		0.4429	0.001276	0.0002318	9.10E-09	0.971156
rs143333049	19	19800289	G		0.04016	0.004958	0.0005947	5.30E-16	0.936316
rs1672991	19	35556659	G		0.9336	0.006755	0.0004552	9.20E-52	0.995622
rs142888784	19	38810744	G		0.02256	-0.008762	0.0008414	7.90E-27	0.818477
rs7248167	19	43960114	C		0.217	-0.001568	0.0002765	8.30E-09	0.983394
rs73036517	19	45744842	G		0.2596	-0.001455	0.0002598	1.90E-08	0.991015
19:50049641_GT_G	19	50049641	G		0.2506	-0.003007	0.0002653	2.50E-29	0.970816
rs12983362	19	52153000	G		0.2647	0.002122	0.0002632	2.30E-15	0.948015
rs73078112	20	5547384	T		0.02722	0.004687	0.0006998	1.30E-11	0.984385
20:33404922_TCA_T	20	33404922	T		0.1866	-0.001665	0.0002926	3.10E-09	0.986813
rs3091842	20	39344272	A		0.04416	0.008099	0.0005697	2.40E-47	0.934223
rs3787267	20	45626851	T		0.6532	-0.001364	0.000241	1.20E-08	0.973764
rs35870583	20	52735238	GT		0.1873	-0.005671	0.0003006	3.40E-82	0.937033
rs185799410	20	57466093	T		0.02602	-0.00399	0.0007238	2.40E-08	0.966718
rs928760	21	35890958	T		0.3027	-0.001714	0.0002475	3.70E-12	0.99857
rs219781	21	37832621	T		0.2632	0.001459	0.0002576	1.40E-08	0.996786
rs5760495	22	25000461	T		0.3566	0.00156	0.0002389	1.30E-11	0.985639
rs28908470	22	43116277	T		0.3232	0.001423	0.0002431	1.60E-09	0.996558

SNP = Rsid, CHR = Chromosome, Position = Physical position (Build 19), N = Sample size, EAF = Effect allele frequency, Beta = Effect size in mmol/L, SE = Standard Error

Supplementary Table II: Look up of previously reported lead variants for serum total calcium in our UKB GWAS

Rsid	Chr	Position	Effect Allele	Reported Beta	SE	Equivalent Beta (mmol/L)	*P*-value	PMID	UKB Beta	UKB SE	UKB *P*-value
rs780094	2	27741237	T	0.017	0.003	0.00425	1.30×10^{-10}	24068962	0.004243	0.0002	1.1×10^{-78}
rs1550532	2	234264848	C	0.018	0.003	0.0045	8.20×10^{-11}	24068962	0.003819	0.0002	1.1×10^{-56}
rs1801725	3	122003757	T	0.071	0.004	0.01775	8.90×10^{-86}	24068962;20705733	0.017199	0.0003	6.0×10^{-589}
rs10491003	10	9368657	T	0.027	0.005	0.00675	4.80×10^{-09}	24068962	0.006183	0.0004	2.4×10^{-57}
rs7481584	11	3029089	A	-0.018	0.003	-0.0045	1.20×10^{-10}	24068962	-0.0023	0.0003	2.5×10^{-21}
rs7336933	13	42559076	A	-0.022	0.004	-0.0055	9.10×10^{-10}	24068962	-0.00325	0.0003	4.2×10^{-25}
rs1570669	20	52207834	A	-0.018	0.003	-0.0045	9.10×10^{-12}	24068962	-0.00269	0.0002	3.8×10^{-31}

Chr = Chromosome, Position = Physical position (Build 37), EAF = Effect allele frequency, Beta = Effect size in original study (mg/dl), SE = Standard Error, UKB Beta = Effect size in our serum total calcium study (mmol/L)

Supplementary Table III: Association between serum albumin-corrected calcium concentration and measures of ventricular depolarisation and repolarisation using Mendelian Randomization

		Inverse-median weighted		Median-Weighted		MR-Egger		MR-PRESSO outlier adjusted	
	SNPs	Beta (95% CI)	*P*-value	Beta (95% CI)	*P*-value	Beta (95% CI)	*P*-value	Beta (95% CI)	*P*-value
QT interval	202	-3.62 (-4.67 – -2.57)	1.60x10-10	-4.90 9-6.31 – -3.49)	$9.23x10^{-12}$	-5.78 (-7.64 – -3.92)	$6.01x10^{-9}$	-4.22 (-5.07 – -3.38)	$1.06x10^{-18}$
JT interval	202	-3.25 (-4.31 – -2.20)	$1.52x10^{-9}$	-4.58 (-5.91 – -3.24)	$3.17x10^{-11}$	-5.66 (7.52 – -3.80)	$1.10x10^{-8}$	-3.80 (-4.64 – -2.95)	$8.89x10^{-16}$
QRS duration	202	-0.39 (-0.69 – -0.08)	0.011	-0.48 (-1.05 – 0.09)	0.10	-0.19 (-0.76 – 0.38)	0.51	-0.41 (-0.73 – -0.10)	0.015

Beta = effect size (ms change per 0.1mmol/L).

Supplementary Table IV: Association between serum total calcium concentration and ECG measures following exclusion of instrumental variants with r2 > 0.001

		Inverse-median weighted		Median-Weighted		MR-Egger		MR-PRESSO outlier adjusted	
	SNPs	Beta (95% CI)	*P*-value	Beta (95% CI)	*P*-value	Beta (95% CI)	*P*-value	Beta (95% CI)	*P*-value
QT interval	129	-3.26 (-4.29 – -2.23)	$4.87x10^{-10}$	-4.92 (-6.09 – -3.75)	$1.62x10^{-16}$	-5.01 (-6.79 – -3.23)	$1.91x10^{-7}$	-3.61 (-4.49 – -2.73)	$5.55x10^{-13}$
JT interval	129	-3.10 (-4.19 – -2.02)	$2.02x10^{-8}$	-4.39 (-5.66 – -3.12)	$1.31x10^{-11}$	-4.84 (-6.72 – -2.95)	$1.64x10^{-6}$	-3.45 (-4.38 – -2.51)	$3.99x10^{-11}$
QRS duration	129	-0.23 (-0.59 – 0.13)	0.21	-0.47 (-0.09 – 1.02)	0.1	-0.22 (-0.85 – 0.41)	0.49	-0.22 (-0.55 – 0.12)	0.21

Beta = effect size (ms change per 0.1mmol/L). For QT and QRS, there were no outliers detected during MR-PRESSO analyses and thus a result is not reported.

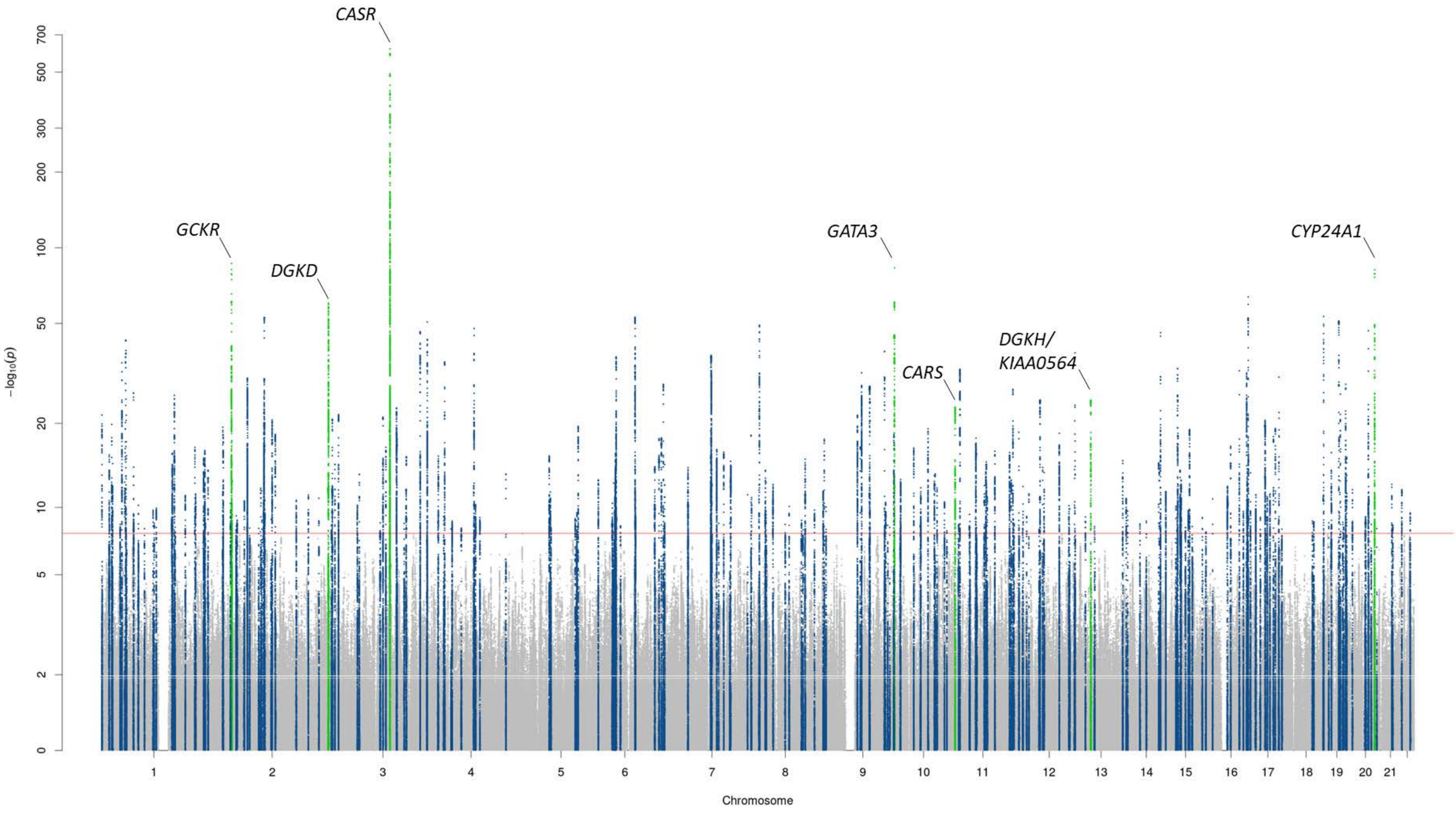

Supplementary Figure I: Manhattan plot – serum total calcium GWAS

P-values expressed as -log10(*P*). Known loci (7) are labelled and highlighted in green. Novel (208) loci are highlighted in blue. Red horizontal line indicates genome wide significance threshold ($5x10^{-8}$).

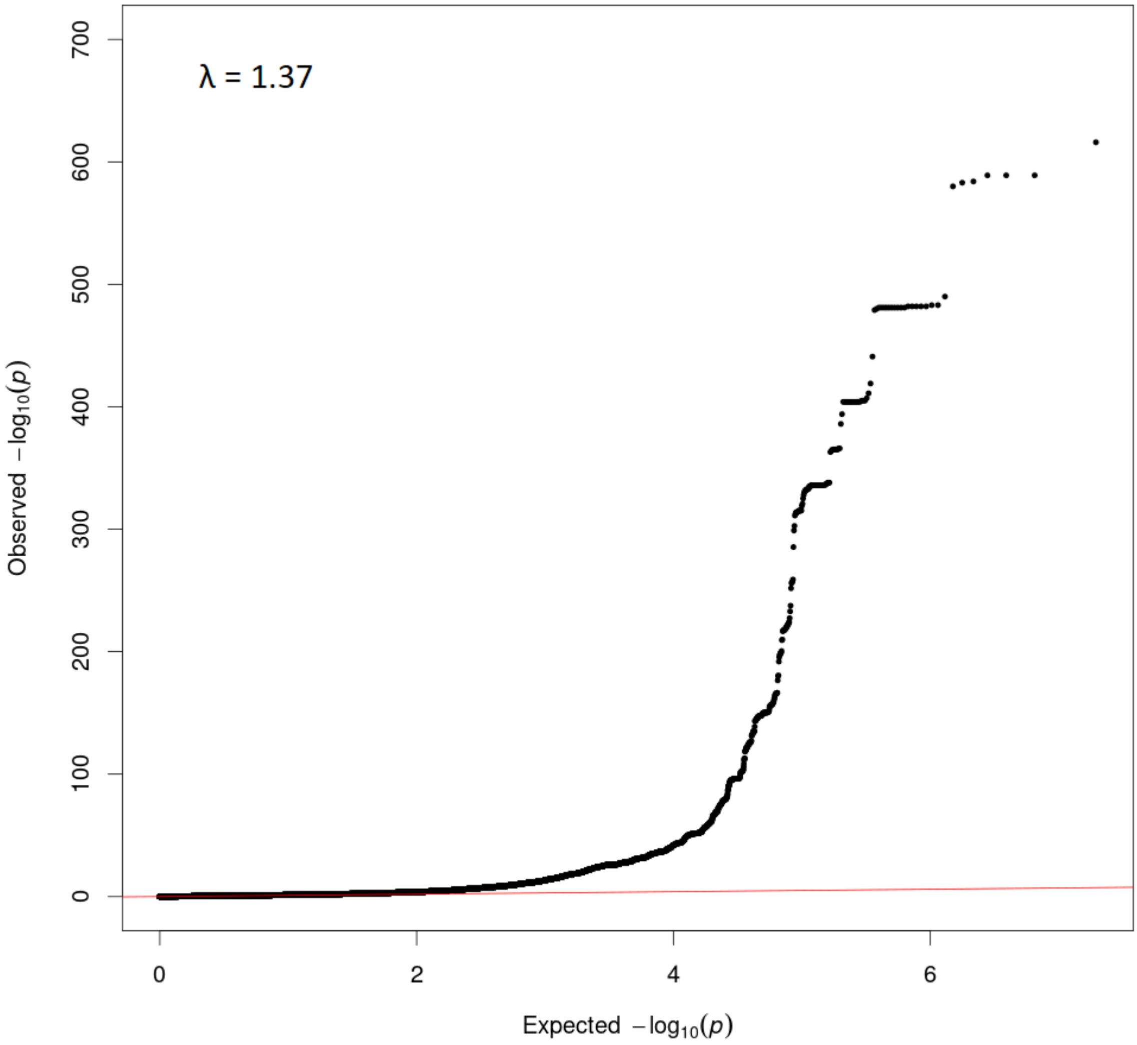

Supplementary Figure II: Quantile-Quantile plot – Serum Total Calcium GWAS
Using linkage disequilibrium score regression (LDSC) using pruned variants to only include those in the HapMap reference panel (N = 1,183,511), the lambda was 1.43, and LD score regression intercept 1.10; suggesting any inflation in lambda is mostly due to polygenicity, rather than confounding bias.

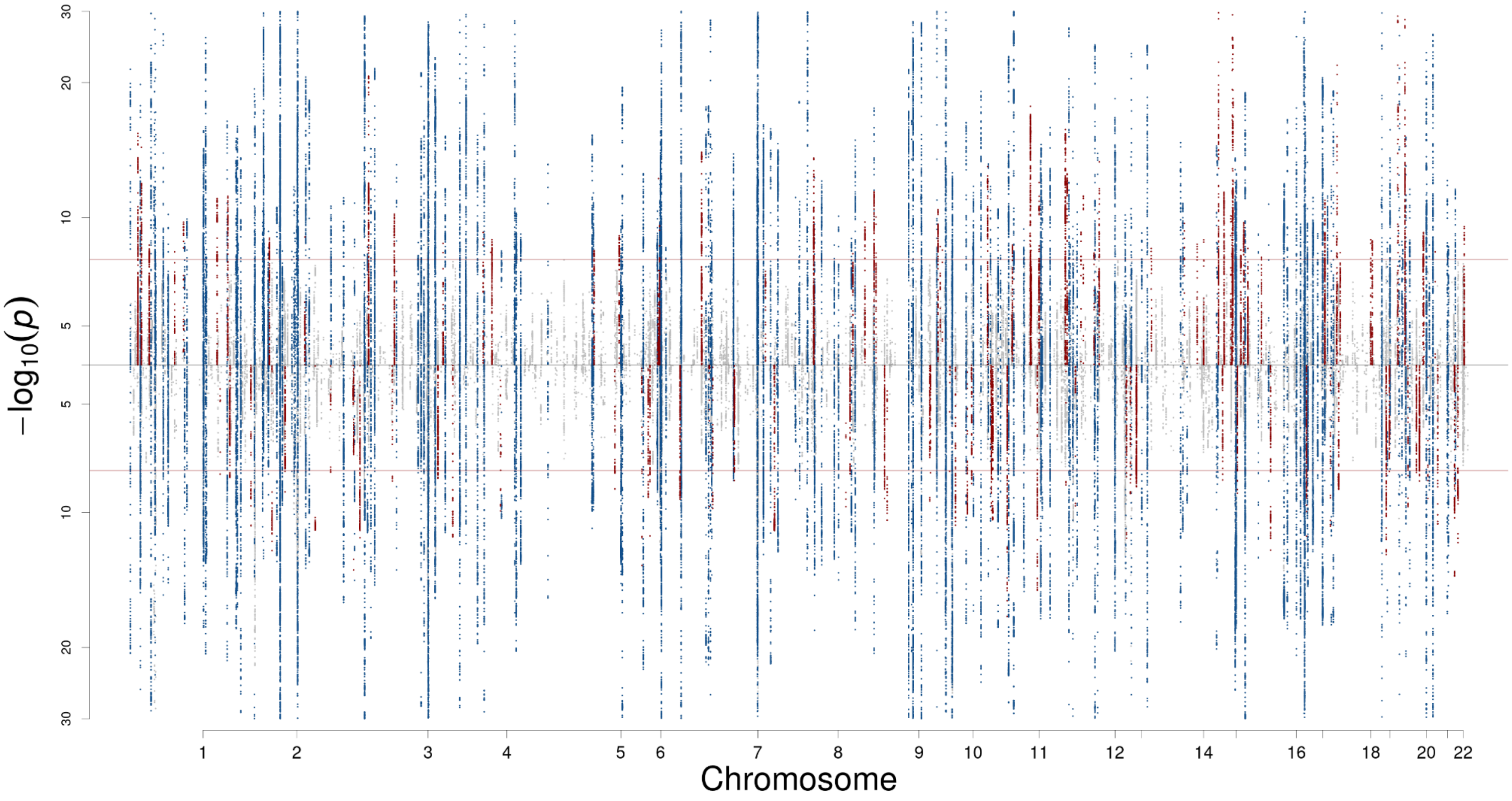

Supplementary Figure III: Miami plot comparing serum total uncorrected calcium and albumin-corrected calcium GWAS results
P-values expressed as -log10(*P*). The Y axis has been limited to include only variants with a *P*-value > 1x10-4 and < 1x10-30, to facilitate comparison. The top panel is GWAS results for total serum calcium and the bottom panel for albumin-corrected calcium. Variants are highlighted in red if within loci which were genome-wide significant in one GWAS but not the other. Variants within loci which were genome-wide significant in both GWAS are highlighted in blue. Red horizontal lines indicate genome wide significance threshold ($5x10^{-8}$).

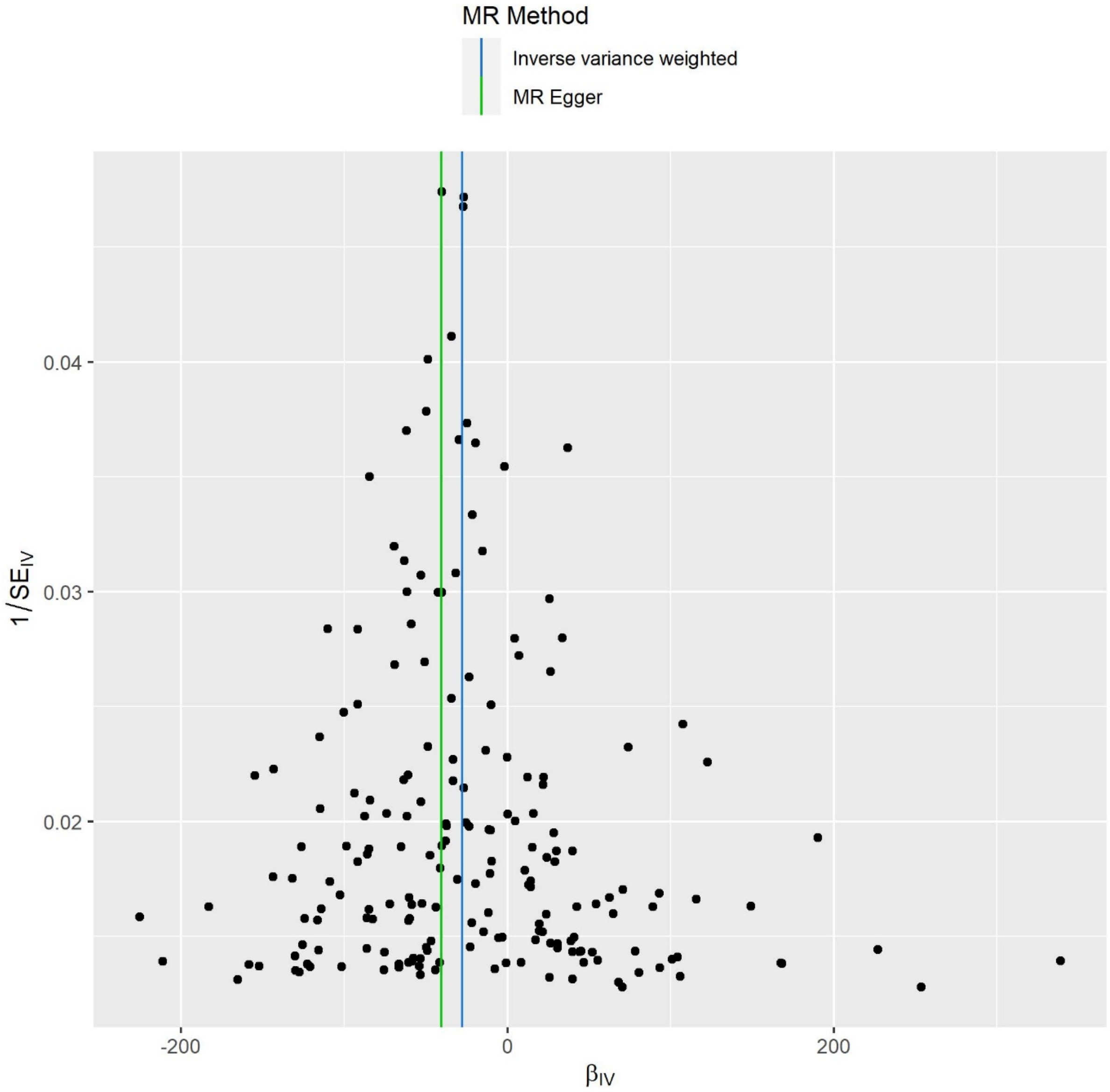

Supplementary Figure IV-A. Funnel plot for mendelian randomisation serum total calcium-QT analyses.
X axis: Effect size of each variant. Y axis: Inverse of the variant's standard error.

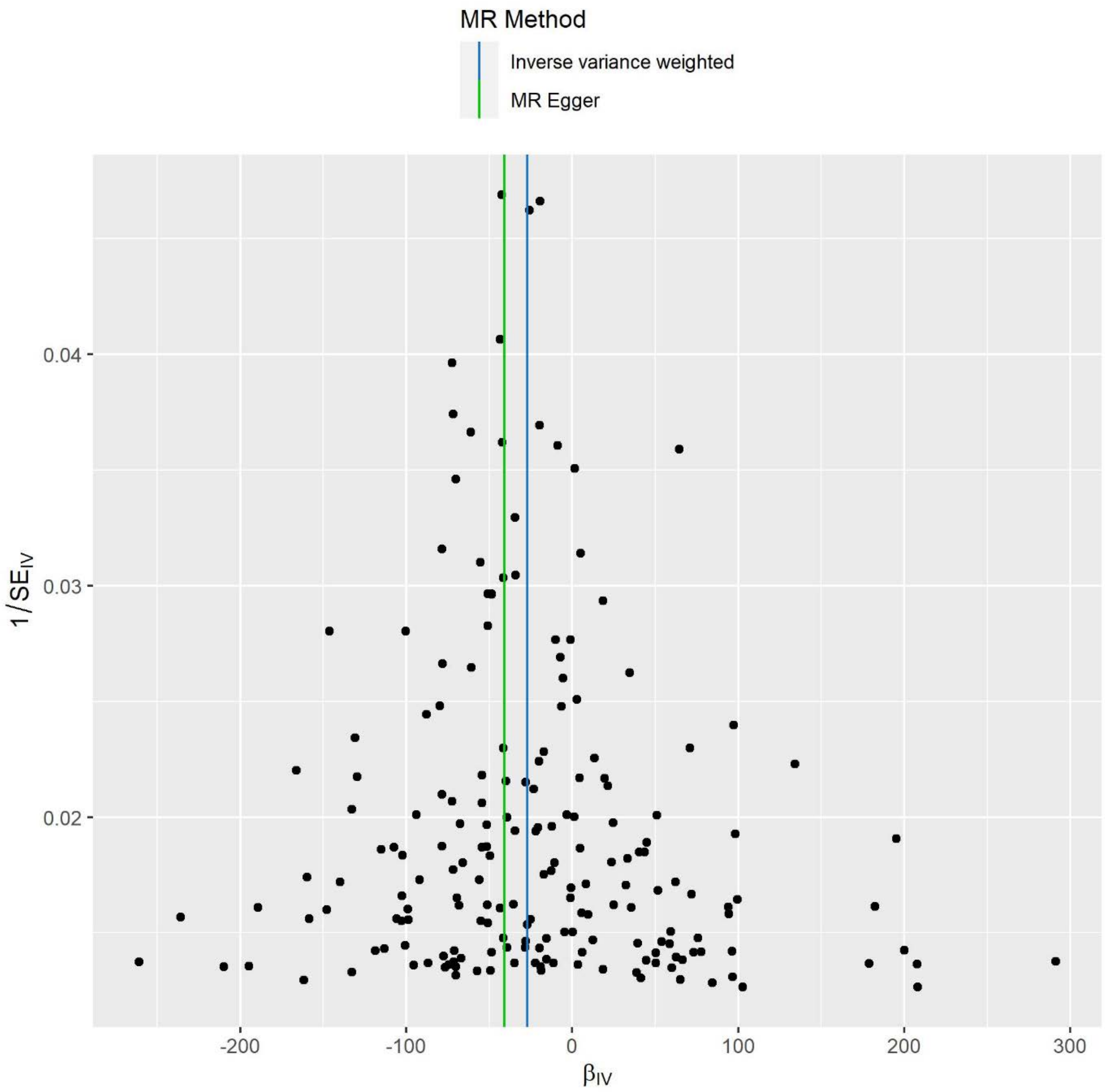

Supplementary Figure IV-B. Funnel plot for mendelian randomisation serum total calcium-JT analyses.
X axis: Effect size of each variant. Y axis: Inverse of the variant's standard error.

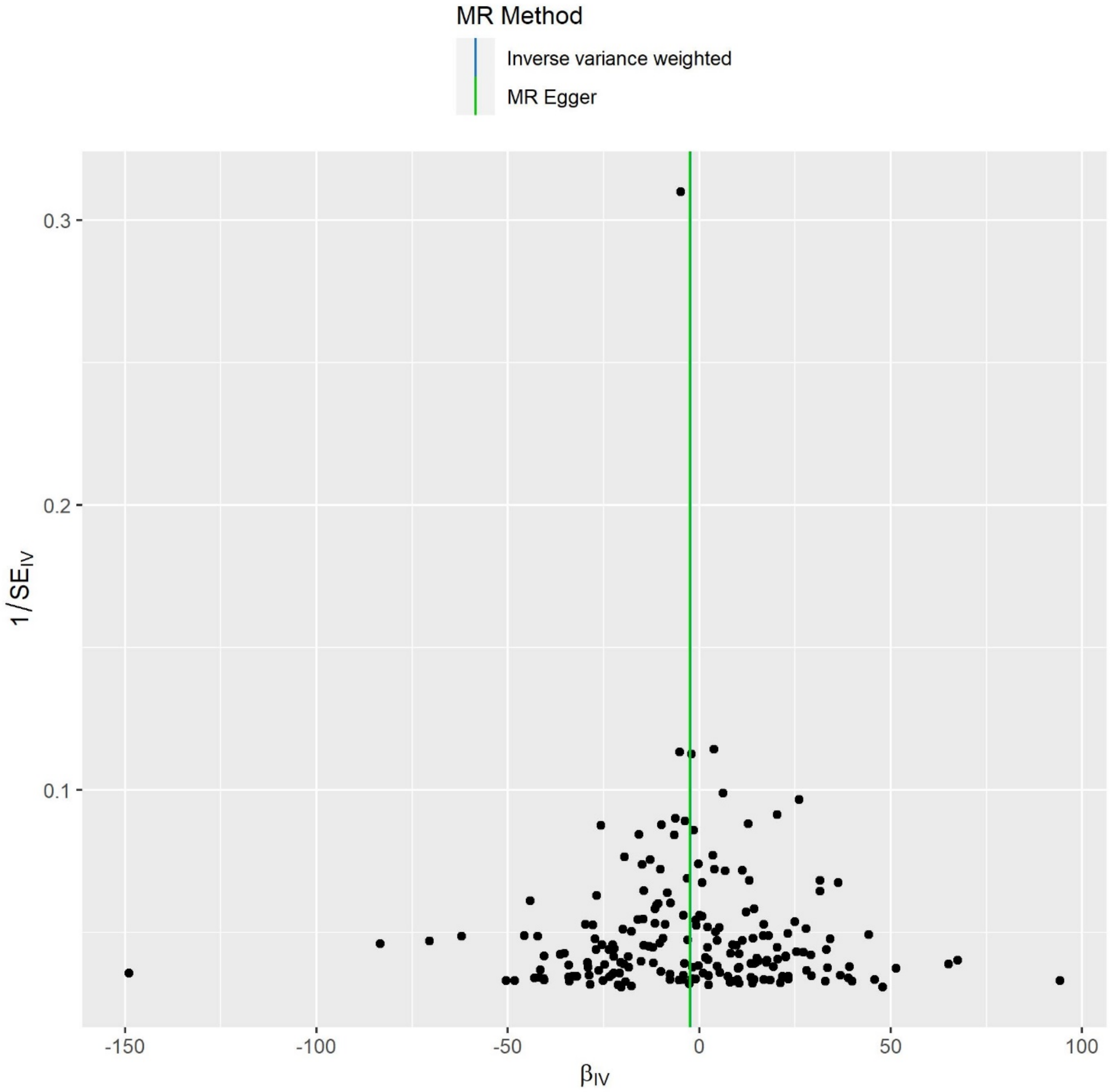

Supplementary Figure IV-C. Funnel plot for mendelian randomisation serum total calcium-QRS analyses.
X axis: Effect size of each variant. Y axis: Inverse of the variant's standard error.
