# Supplementary material for: Genetically Determined Serum Calcium Levels and Markers of Ventricular Repolarization: A Mendelian Randomization Study in the UK Biobank
Source: Circ Genom Precis Med. 2021 Apr 22;14(3):e003231. doi: 10.1161/CIRCGEN.120.003231 (PMC8208093; doi:10.1161/CIRCGEN.120.003231)
Supplement: Supplementary file 2 [file hcg-14-e003231-s002.pdf]

DOI: 10.1161/CIRCGEN.120.003231

Genetically-Determined Serum Calcium Levels and Markers of Ventricular Repolarisation: A Mendelian Randomization Study in the UK Biobank

Running title: *Young et al.; Calcium and Ventricular Repolarisation: A MR study*

William J. Young, MBBS[1,2]; Helen R. Warren, PhD[1,3]; Dennis O. Mook-Kanamori, MD, PhD[4,5]; Julia Ramírez, PhD[1,6]; Stefan van Duijvenboden, PhD[1,6]; Michele Orini, PhD[2,6]; Andrew Tinker, MBBS, PhD[1,3]; Diana van Heemst, PhD[7]; Pier D. Lambiase, BM BCh, PhD[2,6]; J. Wouter Jukema, MD, PhD[8,9]; Patricia B. Munroe, PhD[1,3]; Raymond Noordam, PhD[7]

[1]Clinical Pharmacology Dept, William Harvey Research Inst, Barts & the London School of Medicine & Dentistry, Queen Mary Univ of London; [2]Barts Heart Centre, St Bartholomew's Hospital, Barts Health NHS trust; [3]NIHR Barts Cardiovascular Biomedical Research Unit, Barts & The London School of Medicine & Dentistry, Queen Mary Univ of London, London, UK; [4]Dept of Clinical Epidemiology, [5]Dept of Public Health & Primary Care, [7]Dept of Internal Medicine, [8]Dept of Cardiology, Leiden Univ Medical Center, Leiden, the Netherlands; [6]Institute of Cardiovascular Sciences, Univ of College London, UK; [9]Netherlands Heart Institute, Utrecht, the Netherlands

Circulation: Genomic and Precision Medicine

Correspondence:

William Jon Young MBBS
William Harvey Research Institute
Department of Clinical Pharmacology
Barts and The London School
of Medicine and Dentistry,
Queen Mary University of London,
London, EC1M 6BQ, UK.
Tel: 020 7882 5555
Email: w.young@qmul.ac.uk

Raymond Noordam PhD
Department of Internal Medicine
Section of Gerontology and Geriatrics
Leiden University Medical Center
PO Box 9600
2300 RC Leiden
the Netherlands
Tel: +31 (0)71 526 5213
Email: r.noordam@lumc.nl

Journal Subject Terms: Genetics; Genetic, Association Studies; Electrocardiology (ECG); Electrophysiology; Cardiovascular Disease

This article is published in its accepted form; it has not been copyedited and has not appeared in an issue of the journal. Preparation for inclusion in an issue of *Circulation: Genomic and Precision Medicine* involves copyediting, typesetting, proofreading, and author review, which may lead to differences between this accepted version of the manuscript and the final published version.

Abstract:

Background - Electrocardiographic (ECG) markers of ventricular depolarisation and repolarisation are associated with an increased risk of arrhythmia and sudden cardiac death. Our prior work indicated lower serum calcium concentrations are associated with longer QT and JT intervals in the general population. Here, we investigate whether serum calcium is a causal risk factor for changes in ECG measures using Mendelian Randomization (MR).

Methods - Independent lead variants from a newly performed genome-wide association study (GWAS) for serum calcium in >300,000 European-ancestry participants from UK-Biobank were used as instrumental variables. Two-sample MR analyses were performed to approximate the causal effect of serum calcium on QT, JT and QRS intervals using an inverse-weighted method in 76,226 participants not contributing to the serum calcium GWAS. Sensitivity analyses including MR-Egger, weighted-median estimator, and MR-PRESSO were performed to test for the presence of horizontal pleiotropy.

Results - 205 independent lead calcium-associated variants were used as instrumental variables for MR. A decrease of 0.1 mmol/L serum calcium was associated with longer QT (3.01ms (95% CI 3.99, -2.03) and JT (2.89ms (-3.87, - 1.91) intervals. A weak association was observed for QRS duration (secondary analyses only). Results were concordant in all sensitivity analyses.

Conclusions - These analyses support a causal effect of serum calcium levels on ventricular repolarisation, in a middle-aged population of European-ancestry where serum calcium concentrations are likely stable and chronic. Modulation of calcium concentration may therefore directly influence cardiovascular disease risk.

Key words: Mendelian randomization; electrocardiography; repolarization; calcium; electrocardiographic intervals; ventricular repolarization

Circulation: Genomic and Precision Medicine

This article is published in its accepted form; it has not been copyedited and has not appeared in an issue of the journal. Preparation for inclusion in an issue of *Circulation: Genomic and Precision Medicine* involves copyediting, typesetting, proofreading, and author review, which may lead to differences between this accepted version of the manuscript and the final published version.

DOI: 10.1161/CIRCGEN.120.003231

Nonstandard Abbreviations and Acronyms

BOLT-LMM	BOLT linear mixed model
ECG	Electrocardiogram
EST-UKB	Exercise stress test cohort
GWAS	Genome-wide association study
IMAGING-UKB	Imaging study cohort
InSIDE	INstrument Strength Independent of Direct Effect
IVW	Inverse variance-weighted
LD	Linkage disequilibrium
L-type	Long-lasting
MR	Mendelian Randomization
MRCIEU	MRC Integrative Epidemiology Unit
MR-PRESSO	Mendelian Randomization pleiotropy residual sum and outlier
QQ	Quantile-Quantile
SNP	Single-nucleotide polymorphism
UKB	UK Biobank

American Heart Association.
Circulation: Genomic and Precision Medicine

Introduction

Non-invasive markers of cardiac disease derived from the electrocardiogram (ECG) are associated with major cardiovascular events and reflect underlying abnormalities in cardiac structure and electrical conduction[1-4]. Abnormal action potential duration and amplification of the spatial dispersion of repolarisation, coupled with early after depolarisations inducing triggered activity is an important mechanism of ventricular arrhythmia, specifically torsades de pointes tachycardia[5, 6]. Prolongation of the QT interval, a marker of the time needed for ventricular repolarisation and depolarisation, has consistently been associated with adverse outcomes, including ventricular arrhythmia and sudden cardiac death[7-9]. QRS duration (time point from QRS onset to offset) is specific for ventricular depolarisation while the JT interval is specific for ventricular repolarisation spanning the interval from QRS offset to T-wave end. Multiple factors may influence these ECG markers and thus the potential for arrhythmia,

This article is published in its accepted form; it has not been copyedited and has not appeared in an issue of the journal. Preparation for inclusion in an issue of *Circulation: Genomic and Precision Medicine* involves copyediting, typesetting, proofreading, and author review, which may lead to differences between this accepted version of the manuscript and the final published version.

including mutations in genes encoding ion channels and their accessory proteins (e.g *KCNQ1* and *KCNE1*) and iatrogenic causes due to off target effects by medication (e.g., cancer therapeutics and psychotropics)[10-12].

The different phases of the cardiac action potential are caused by the (inward and outward) movement of different ions across the membrane of the cardiac cells. Serum electrolyte concentrations are associated with alterations in ECG derived indices of cardiac electrophysiological activity. Historically, studies have focused on the effects of electrolytes in clinical populations often with serum electrolyte concentrations significantly outside of the normal range and/or rapid and acute changes in their concentration[13, 14]. We recently published the results of a large meta-analysis of cross-sectional data including 153,014 unselected individuals, investigating the association of serum electrolyte levels with ECG-derived indices[15]. One of the key findings was an association between lower serum calcium and longer QT (2.23 ms per 0.1 mmol/L) and JT (2.27 ms per 0.1 mmol/L) intervals but not with QRS duration. The lack of a calcium-QRS duration association suggested serum calcium specifically affects ventricular repolarisation. However, given the observational and cross-sectional nature of the study, and the limited number of considered confounders, we were unable to determine whether these observations were causal.

American Heart Association.
Circulation: Genomic and Precision Medicine

Mendelian randomization (MR), in which genetic variants significantly associated with an exposure are used to estimate causal effects of that exposure on outcomes of interest[16-18], has been widely used to assess causality in observational settings. MR overcomes the main limitations of observational studies, notably reverse causation and residual confounding[19]. Previous genome-wide association studies (GWAS) for serum calcium have identified associated variants, and have been leveraged before in MR studies for cardiovascular disease risk[20-23].

This article is published in its accepted form; it has not been copyedited and has not appeared in an issue of the journal. Preparation for inclusion in an issue of *Circulation: Genomic and Precision Medicine* involves copyediting, typesetting, proofreading, and author review, which may lead to differences between this accepted version of the manuscript and the final published version.

However, due to the relatively small sample sizes of these GWAS which limited the number of associations identified, the genetic instruments included in MR analyses explained only a small proportion of the variance of calcium (~0.9%) [24, 25]. The release of biochemical data in UK Biobank (UKB) permits the identification of additional genetic variants for serum calcium in larger samples increasing the number of variants and consequently increasing the power of an MR study[26, 27]. In this study, we performed a new GWAS on serum total calcium and used the independent lead variants as instrumental variables to assess potential causality of the association between lower serum calcium and prolongation of QT and JT intervals in UKB, including QRS duration as a negative control.

American Heart Association.

Methods

Anonymized clinical, genotype and ECG data were obtained from UKB[27]. The UKB study has approval from the NHS North West Multi-Centre Research Ethics Committee (ref 11/NW/0382) and participating studies provided informed consent. Any data generated by this study will be returned to UKB in accordance with researcher obligations, to be made available for further research. Full methods are available in Supplementary Methods, Data Supplement and also summarized in Figure 1.

Circulation: Genomic and Precision Medicine

Results

Calcium GWAS

We identified 208 independent lead variants (201 from novel loci) associated with serum total calcium concentration at genome-wide significance level ($P<5x10^{-8}$) (Supplementary Table 1, Data Supplement). A Manhattan plot and QQ plot are shown in Supplementary Figures 1 and 2

This article is published in its accepted form; it has not been copyedited and has not appeared in an issue of the journal. Preparation for inclusion in an issue of *Circulation: Genomic and Precision Medicine* involves copyediting, typesetting, proofreading, and author review, which may lead to differences between this accepted version of the manuscript and the final published version.

respectively in the Data Supplement. The percentage variance of total serum calcium explained by variants included in this MR study was 5.8% (compared with 0.9% for previously reported variants)[24]. Previously reported variants associated with serum calcium showed the same direction of effect and similar effect size estimates (Supplementary Table 2, Data Supplement). There were 208 independent lead GWS variants identified in the albumin-corrected calcium GWAS, of which 151 were in loci overlapping with those reported in the uncorrected calcium GWAS at *P*-value < $5x10^{-8}$. (Supplementary Figure 3, Data Supplement). The correlation between results of GWS loci between the original vs the albumin-corrected GWAS was $r^2 = 0.88$ for the beta estimates and $r^2 = 0.55$ for the *P*-values (spearman rank coefficient). Following exclusion of palindromic SNPs with intermediate allele frequencies, 205 and 202 variants for total serum calcium and albumin-corrected calcium respectively, were included in MR analyses.

American Heart Association.
Circulation: Genomic and Precision Medicine

Mendelian randomisation analyses

Primary analysis - IVW

Study characteristics for individuals included in each ECG cohort specific GWAS and subsequently combined in the meta-analysis, and the calcium GWAS are shown in Table 1. A total of 76,266 participants were included with a median age of 61 (interquartile range: 54-66) years and 53.1% were women.

The results for the estimated causal effect of total serum calcium on the ECG measures are shown in Table 2. Using the IVW model, a genetically-determined 0.1 mmol/L decrease in serum total calcium was associated with a 3.01 ms (95% CI -3.99, -2.03) longer QT interval and a 2.89 ms (-3.87, -1.91) longer JT interval. No association was found with QRS duration (-0.20 ms (-0.49, 0.10). The results for albumin-corrected calcium were similar showing the strongest

This article is published in its accepted form; it has not been copyedited and has not appeared in an issue of the journal. Preparation for inclusion in an issue of *Circulation: Genomic and Precision Medicine* involves copyediting, typesetting, proofreading, and author review, which may lead to differences between this accepted version of the manuscript and the final published version.

association with QT and JT intervals, but a weak association with QRS duration was observed (-0.39 ms (-0.69, -0.08)) (Supplementary Table 3, Data Supplement).

Sensitivity analyses

Genetically-determined lower serum calcium concentrations were consistently associated with longer QT and JT intervals across sensitivity analyses using weighted median estimator, MR-Egger and MR-PRESSO methods, with similar or stronger effect sizes as using the IVW model (Table 2, Supplementary Table 3). Furthermore, we did not observe that any of the intercepts with MR-Egger deviated significantly from zero (P-values>0.05), indicating no evidence of bias from pleiotropy. The results were similar after exclusion of instrumental variants using a more stringent r^2 threshold (> 0.001), (Supplementary Table 4, Data Supplement).

Similar results were identified after exclusion of the variant mapped to *CASR*, a locus, which is a major genetic determinant of serum calcium concentration[21]. Scatter plots for serum total calcium analyses are presented in Figures 2A-C for each ECG measure. Funnel plots did not indicate any directional horizontal pleiotropy (Supplementary Figures 4A-C, Data Supplement).

American Heart Association.
Circulation: Genomic and Precision Medicine

Discussion

This study utilises MR to demonstrate the causal inverse relationship between serum calcium concentration and longer QT and JT intervals in UKB, a large middle-aged European ancestry population. This inverse relationship was consistent across all sensitivity analyses. These results along with the absence of a clinically relevant association with QRS duration due to its very small effect size, collectively suggest that a genetically-predicted lower serum calcium is a causal contributor primarily for increasing ventricular repolarization time in a population where serum calcium concentration exposure is likely stable and chronic. They also highlight the utility

This article is published in its accepted form; it has not been copyedited and has not appeared in an issue of the journal. Preparation for inclusion in an issue of *Circulation: Genomic and Precision Medicine* involves copyediting, typesetting, proofreading, and author review, which may lead to differences between this accepted version of the manuscript and the final published version.

of MR in the investigation of clinically relevant variables and their contribution, to specific time points in ventricular cardiac electrophysiology.

It is well recognised that extremes of both hypocalcaemia and hypercalcaemia in clinical cohorts result in prolongation and shortening of ventricular repolarisation respectively[28]. However, there has previously been limited study of the influence of stable calcium concentrations in population-based studies. We previously reported an inverse association between serum total calcium concentration and QT and JT intervals in a large meta-analysis of observational studies with over 150K unselected individuals[15]. Specifically, we observed a 2.23 ms longer QT interval and 2.27 ms longer JT interval per 0.1 mmol/L decrease in serum calcium, in the absence of a limited number of considered confounding factors[15]. These effect size estimates are similar to those obtained in this MR study, using individuals from UKB. It should be noted that UKB was not included in our previous observational meta-analysis study and is thus an independent cohort. When comparing the MR results of serum uncorrected calcium with the secondary analysis using albumin-corrected calcium, our findings were very similar with strong associations identified with QT and JT intervals. The marginal association between serum albumin-corrected calcium and QRS duration was considered not clinically relevant and anticipated given some overlap exists between the genetic contributions of QRS and QT/JT intervals[29].

Previous randomised control and crossover trials estimated an increase in serum total calcium of 0.07 – 0.13 mmol/L approximately 4 hours after ingestion of calcium carbonate (500mg)[30, 31]. Thus, the results of this study suggest oral calcium supplementation could temporarily decrease the QT interval by 2.11 – 3.91 ms. As the effect of oral calcium supplementation on serum total calcium concentration is small, we would expect no direct

Circulation: Genomic and Precision Medicine

This article is published in its accepted form; it has not been copyedited and has not appeared in an issue of the journal. Preparation for inclusion in an issue of *Circulation: Genomic and Precision Medicine* involves copyediting, typesetting, proofreading, and author review, which may lead to differences between this accepted version of the manuscript and the final published version.

DOI: 10.1161/CIRCGEN.120.003231

clinical benefits. However, the results of this study suggest further research into the effects of serum calcium concentration on arrhythmogenesis is warranted and calcium variants could be considered for inclusion in genetic risk score models for risk prediction. This may be of particular importance in patient sub-groups such as endocrinology disorders affecting calcium homeostasis, concurrent use of medication which prolong the QT interval, and in the context of other co-morbidities where a substrate exists for ventricular arrhythmia such as ischemic heart disease, cardiomyopathies or channelopathies[12, 32].

Although an inverse relationship between calcium and markers of ventricular repolarisation were identified in this study, associations between higher serum calcium concentrations and increased cardiovascular disease risk including myocardial infarction, stroke and cardiovascular mortality have been reported in individual epidemiological studies, meta-analyses and some randomised control trials[33-35]. These observations are present at serum calcium concentrations within the normal reference range (association at high-normal concentrations). Thus, there is interest in the use of serum calcium levels in the assessment of cardiovascular risk. To date, six MR studies have been performed evaluating the effect of calcium on cardiovascular outcomes using seven independent variants identified from a previous serum calcium meta-analysis (N ~61,000)[24]. Despite the small percentage variance of calcium explained by these variants (~0.9%), a significant association was identified between serum calcium and coronary artery disease and myocardial infarction, a finding recently replicated in a MR-PheWAS performed in UK Biobank (OR 1.99 for myocardial infarction per 0.25 mmol/L increase in genetically predicted serum calcium, CI: 1.17 – 3.39)[20, 36, 37]. For atrial fibrillation, an MR study identified no significant association in the main analyses[21]. However directional pleiotropy was identified and in MR-Egger analyses, an association was observed (OR 1.30 per

American Heart Association.
Circulation: Genomic and Precision Medicine

This article is published in its accepted form; it has not been copyedited and has not appeared in an issue of the journal. Preparation for inclusion in an issue of *Circulation: Genomic and Precision Medicine* involves copyediting, typesetting, proofreading, and author review, which may lead to differences between this accepted version of the manuscript and the final published version.

0.25 mmol/L increase, CI 1.05 – 1.59) driven by a single variant in the *CASR* locus. This variant out of those included as instrumental variables, explained 0.5% of the variance of serum calcium. Significant associations have not been observed with heart failure (as an endpoint after myocardial infarction) or stroke risk[22, 23]. Additionally, despite calcium supplementation being common in the general population with the intention to reduce the risk of fractures, an association between life-long calcium levels and risk of fracture was not observed in a previous MR study[38]. However, these studies may have been limited by the low variation of calcium explained by variants included in the MR analyses, despite having large sample sizes for testing these clinical outcomes[39].

Despite showing evidence for a causal association between lower serum calcium and longer QT and JT intervals, this study does not provide information on the biological mechanisms involved, which remain uncertain. In animal models, the duration of phase II of the cardiac action potential is determined by the inactivation of voltage-gated long-lasting (L-type) calcium channels, which are dependent on calcium entering these channels and their release from the sarcoplasmic reticulum[28, 40]. Higher extracellular calcium concentrations increase L-type calcium channel inactivation which in turn reduces phase II of the action potential and the inverse is present in lower calcium concentration states, as identified in a more recent in-silico theoretical study using a human ventricular myocyte model[41]. These mechanisms could explain the associations observed in our study between serum calcium and ventricular repolarisation.

American Heart Association.
Circulation: Genomic and Precision Medicine

Strengths and limitations

The present study performed a new serum calcium GWAS to increase the number of genetic instrumental variables and to increase the variance explained to perform a more statistically powerful MR analysis. Furthermore, two-sample MR studies assume the two samples (exposure

This article is published in its accepted form; it has not been copyedited and has not appeared in an issue of the journal. Preparation for inclusion in an issue of *Circulation: Genomic and Precision Medicine* involves copyediting, typesetting, proofreading, and author review, which may lead to differences between this accepted version of the manuscript and the final published version.

and outcome) were performed in different individuals from the same source population. By design, we performed the new calcium GWAS in individuals not contributing to the QT/JT/QRS intervals GWASs ensuring this assumption was met.

UKB is a densely phenotyped cohort and participants are generally healthy compared with the general UK population. Additionally, this study was conducted only in individuals of European ancestry due to a limited sample size available for other ancestries. Therefore, these results may not be extrapolated to population groups of non-European ancestry or within high-risk clinical cohorts such as post-myocardial infarction or channelopathies showing a mendelian pattern of inheritance.

Conclusion

In summary, this MR study indicates that genetically-determined lower serum calcium concentrations are causally associated with longer ventricular repolarization time in a middle-aged population where serum calcium concentration exposure is likely stable and chronic. Modulation of calcium concentration may therefore directly influence cardiovascular disease risk. Additionally, we have shown that the power of MR studies can be harnessed to improve our understanding of cardiac electrophysiology and a similar approach could be considered using other clinically relevant exposures.

Circulation: Genomic and Precision Medicine

Acknowledgments: This research has been conducted using the UKB Resource (application 8256 - Understanding genetic influences in the response of the cardiac electrical system to exercise)

Sources of Funding: WJY is supported by a Medical Research Council grant MR/R017468/1. This research has been conducted using the UKB Resource (application 8256 - Understanding genetic influences in the response of the cardiac electrical system to exercise) and is supported

This article is published in its accepted form; it has not been copyedited and has not appeared in an issue of the journal. Preparation for inclusion in an issue of *Circulation: Genomic and Precision Medicine* involves copyediting, typesetting, proofreading, and author review, which may lead to differences between this accepted version of the manuscript and the final published version.

by MRC grant MR/N025083/1. WJY, HRW, JR, AT, PDL and PBM acknowledge the NIHR Cardiovascular Biomedical Centre at Barts and The London, Queen Mary University of London. PDL is supported by UCL/UCLH Biomedicine NIHR, Barts Heart Centre Biomedical Research Centre. JR acknowledges support from the European Union's Horizon 2020 research and innovation programme under the Marie Sklodowska-Curie grant agreement No.786833.

Disclosures: Dennis Mook-Kanamori MD PhD is a part time research consultant at Metabolon, Inc. All other co-authors declare to have no conflict of interest to declare

Supplemental Materials:
Supplemental Methods
Supplemental Tables I-IV
Supplemental Figures I-IV
References [51-59]

Circulation: Genomic and Precision Medicine

References:

1. Hayıroğlu M, Lakhani I, Tse G, Çınar T, Çinier G, Tekkeşin A. In-hospital Prognostic Value of Electrocardiographic Parameters Except ST-Segment Changes in Acute Myocardial Infarction: Literature Review and Future Perspectives. *Heart Lung Circ*. 2020;29:1603-1612.

2. Enriquez A, Riley M, Marchlinski F. Noninvasive clues for diagnosing ventricular tachycardia mechanism. *J Electrocardiol*. 2018;51:163-169.

3. Holkeri A, Eranti A, Haukilahti MAE, Kerola T, Kentta TV, Tikkanen JT, Anttonen O, Noponen K, Seppanen T, Rissanen H, et al. Predicting sudden cardiac death in a general population using an electrocardiographic risk score. *Heart*. 2020;106:427-433.

4. Kristensen SL, Castagno D, Shen L, Jhund PS, Docherty KF, Rorth R, Abrahim WT, Desai AS, Dickstein K, Rouleau JL, et al. Prevalence and incidence of intraventricular conduction delays and outcomes in patients with heart failure and reduced ejection fraction: Insights from PARADIGM-HF and ATMOSPHERE. *Eur J Heart Fail*. 2020;22:2370-2379.

5. Antzelevitch C. Cardiac repolarization. The long and short of it. *Europace*. 2005;7 Suppl 2:3-9.

6. Koplan BA, Stevenson WG. Ventricular tachycardia and sudden cardiac death. *Mayo Clin Proc*. 2009;84:289-297.

This article is published in its accepted form; it has not been copyedited and has not appeared in an issue of the journal. Preparation for inclusion in an issue of *Circulation: Genomic and Precision Medicine* involves copyediting, typesetting, proofreading, and author review, which may lead to differences between this accepted version of the manuscript and the final published version.

7. Straus SM, Kors JA, De Bruin ML, van der Hooft CS, Hofman A, Heeringa J, Deckers JW, Kingma JH, Sturkenboom MC, Stricker B, et al. Prolonged QTc interval and risk of sudden cardiac death in a population of older adults. *J Am Coll Cardiol*. 2006;47:362-367.

8. Beinart R, Zhang Y, Lima JA, Bluemke DA, Soliman EZ, Heckbert SR, Post WS, Guallar E, Nazarian S. The QT interval is associated with incident cardiovascular events: the MESA study. *J Am Coll Cardiol*. 2014;64:2111-2119.

9. Schwartz PJ, Wolf S. QT interval prolongation as predictor of sudden death in patients with myocardial infarction. *Circulation*. 1978;57:1074-1077.

10. Bohnen MS, Peng G, Robey SH, Terrenoire C, Iyer V, Sampson KJ, Kass RS. Molecular Pathophysiology of Congenital Long QT Syndrome. *Physiol Rev*. 2017;97:89-134.

11. Porta-Sánchez A, Gilbert C, Spears D, Amir E, Chan J, Nanthakumar K, Thavendiranathan P. Incidence, Diagnosis, and Management of QT Prolongation Induced by Cancer Therapies: A Systematic Review. *J Am Heart Assoc*. Dec 2017;6.

12. Beach SR, Celano CM, Sugrue AM, Adams C, Ackerman MJ, Noseworthy PA, Huffamn JC. QT Prolongation, Torsades de Pointes, and Psychotropic Medications: A 5-Year Update. *Psychosomatics*. 2018;59:105-122.

13. Ahmed R, Kiya F, Kitano K, Takagi H, Hashiba K. Effects of combined changes in serum calcium and potassium on QT interval. A study by Holter electrocardiographic monitoring during hemodialysis. *Jpn Heart J*. 1987;28:813-828.

14. Surawicz B. Relationship between electrocardiogram and electrolytes. *Am Heart J*. 1967;73:814-834.

15. Noordam R, Young WJ, Salman R, Kanters JK, van den Berg ME, van Heemst D, Lin HJ, Barreto SM, Biggs, ML, Biino G, et al. Effects of Calcium, Magnesium, and Potassium Concentrations on Ventricular Repolarization in Unselected Individuals. *J Am Coll Cardiol*. 2019;73:3118-3131.

16. Lawlor DA. Commentary: Two-sample Mendelian randomization: opportunities and challenges. *Int J Epidemiol*. 2016;45:908-15.

17. Lawlor DA, Harbord RM, Sterne JA, Timpson N, Davey Smith G. Mendelian randomization: using genes as instruments for making causal inferences in epidemiology. *Statistics in medicine*. 2008;27:1133-1163.

18. Hemani G, Zheng J, Elsworth B, Wade KH, Haberland V, Baird D, Laurin C, Burgess S, Bowden J, Langdon R, et al. The MR-Base platform supports systematic causal inference across the human phenome. *Elife*. 2018;7.

American Heart Association.
Circulation: Genomic and Precision Medicine

This article is published in its accepted form; it has not been copyedited and has not appeared in an issue of the journal. Preparation for inclusion in an issue of *Circulation: Genomic and Precision Medicine* involves copyediting, typesetting, proofreading, and author review, which may lead to differences between this accepted version of the manuscript and the final published version.

19. Emdin CA, Khera AV, Kathiresan S. Mendelian Randomization. *JAMA*. 2017;318:1925-1926.

20. Xu L, Lin SL, Schooling CM. A Mendelian randomization study of the effect of calcium on coronary artery disease, myocardial infarction and their risk factors. *Sci Rep*. 2017;7:42691.

21. Larsson SC, Drca N, Michaelsson K. Serum Magnesium and Calcium Levels and Risk of Atrial Fibrillation. *Circ Genom Precis Med*. 2019;12:e002349.

22. Larsson SC, Traylor M, Burgess S, Boncoraglio GB, Jern C, Michaëlsson K, Markus HS; MEGASTROKE project of the International Stroke Genetics Consortium. Serum magnesium and calcium levels in relation to ischemic stroke: Mendelian randomization study. *Neurology*. 2019;92:e944-e950.

23. Helte E, Åkesson A, Larsson SC. Assessing Causality in Associations of Serum Calcium and Magnesium Levels With Heart Failure: A Two-Sample Mendelian Randomization Study. *Front Genet*. 2019;10:1069.

24. O'Seaghdha CM, Wu H, Yang Q, Kapur K, Guessous I, Zuber AM, Köttgen A, Stoudmann C, Teumer A, Kutalik Z, et al. Meta-analysis of genome-wide association studies identifies six new Loci for serum calcium concentrations. *PLoS Genet*. 2013;9:e1003796.

25. O'Seaghdha CM, Yang Q, Glazer NL, Leak TS, Dehghan A, Smith AV, Kao WH, Lohman K, Hwang SJ, Johnson AD, et al. Common variants in the calcium-sensing receptor gene are associated with total serum calcium levels. *Hum Mol Genet*. Nov 2010;19:4296-4303.

26. Biobank U. Serum biochemistry manual. https://www.ukbiobank.ac.uk/uk-biobank-biomarker-panel/.

27. Bycroft C, Freeman C, Petkova D, Band G, Elliott LT, Sharp K, Motyer A, Vukcevic D, Delaneau O, O'Connell J, et al. The UK Biobank resource with deep phenotyping and genomic data. *Nature*. 10 2018;562:203-209.

28. Kass RS, Tsien RW. Control of action potential duration by calcium ions in cardiac Purkinje fibers. *J Gen Physiol*. 1976;67:599-617.

29. Sotoodehnia N, Isaacs A, de Bakker PI, Dorr M, Newton-Cheh C, Nolte IM, van der Harst P, Müller M, Eijgelsheim M, Alonso A, et al. Common variants in 22 loci are associated with QRS duration and cardiac ventricular conduction. *Nat Genet*. 2010;42:1068-1076.

30. Heaney RP, Dowell MS, Bierman J, Hale CA, Bendich A. Absorbability and cost effectiveness in calcium supplementation. *J Am Coll Nutr*. 2001;20:239-246.

31. Bristow SM, Gamble GD, Stewart A, Kalluru R, Horne AM, Reid IR. Acute effects of calcium citrate with or without a meal, calcium-fortified juice and a dairy product meal on serum calcium and phosphate: a randomised cross-over trial. *Br J Nutr*. 2015;113:1585-1594.

This article is published in its accepted form; it has not been copyedited and has not appeared in an issue of the journal. Preparation for inclusion in an issue of *Circulation: Genomic and Precision Medicine* involves copyediting, typesetting, proofreading, and author review, which may lead to differences between this accepted version of the manuscript and the final published version.

DOI: 10.1161/CIRCGEN.120.003231

32. Brown SJ, Ruppe MD, Tabatabai LS. The Parathyroid Gland and Heart Disease. *Methodist Debakey Cardiovasc J*. 2017;13:49-54.

33. Bolland MJ, Grey A, Avenell A, Gamble GD, Reid IR. Calcium supplements with or without vitamin D and risk of cardiovascular events: reanalysis of the Women's Health Initiative limited access dataset and meta-analysis. *BMJ*. 2011;342:d2040.

34. Rohrmann S, Garmo H, Malmström H, Hammar N, Jungar I, Walldius G, Van Hemelrijck M. Association between serum calcium concentration and risk of incident and fatal cardiovascular disease in the prospective AMORIS study. *Atherosclerosis*. 2016;251:85-93.

35. Reid IR, Gamble GD, Bolland MJ. Circulating calcium concentrations, vascular disease and mortality: a systematic review. *J Intern Med*. 2016;279:524-540.

36. Zhou A, Morris HA, Hyppönen E. Health effects associated with serum calcium concentrations: evidence from MR-PheWAS analysis in UK Biobank. *Osteoporos Int*. 2019;30:2343-2348.

37. Larsson SC, Burgess S, Michaëlsson K. Association of Genetic Variants Related to Serum Calcium Levels With Coronary Artery Disease and Myocardial Infarction. *JAMA*. 2017;318:371-380.

38. Cerani A, Zhou S, Forgetta V, Morris JA, Trajanoska K, Rivadeneira F, Larsson SC, Michaëlsson K, Richards JB. Genetic predisposition to increased serum calcium, bone mineral density, and fracture risk in individuals with normal calcium levels: mendelian randomisation study. *BMJ*. 2019;366:l4410.

39. von Hinke Kessler Scholder S, Smith GD, Lawlor DA, Propper C, Windmeijer F. Mendelian randomization: the use of genes in instrumental variable analyses. *Health Econ*. 2011;20:893-896.

40. Linz KW, Meyer R. Control of L-type calcium current during the action potential of guinea-pig ventricular myocytes. *J Physiol*. 1998;513:425-442.

41. Grandi E, Pasqualini FS, Pes C, Corsi C, Zaza A, Severi S. Theoretical investigation of action potential duration dependence on extracellular Ca2+ in human cardiomyocytes. *J Mol Cell Cardiol*. 2009;46:332-342.

42. Loh PR, Tucker G, Bulik-Sullivan BK, Vilhjálmsson BJ, Finucane HK, Salem RM, Chasman DI, Ridker PM, Neale BM, Berger B, et al. Efficient Bayesian mixed-model analysis increases association power in large cohorts. *Nat Genet*. 2015;47:284-90.

43. Correcting the calcium. *Br Med J*. 1977;1:598.

Circulation: Genomic and Precision Medicine

This article is published in its accepted form; it has not been copyedited and has not appeared in an issue of the journal. Preparation for inclusion in an issue of *Circulation: Genomic and Precision Medicine* involves copyediting, typesetting, proofreading, and author review, which may lead to differences between this accepted version of the manuscript and the final published version.

44. Payne RB, Little AJ, Williams RB, Milner JR. Interpretation of serum calcium in patients with abnormal serum proteins. *Br Med J*. Dec 1973;4:643-646.

45. Winkler TW, Day FR, Croteau-Chonka DC, Wood AR, Locke AE, Mägi R, Ferreira T, Fall T, Graff M, Justice AE, et al. Quality control and conduct of genome-wide association meta-analyses. *Nat Protoc*. 2014;9:1192-1212.

46. Bulik-Sullivan BK, Loh PR, Finucane HK, Ripke S, Yang J; Schizophrenia Working Group of the Psychiatric Genomics Consortium, Patterson N, Daly MJ, Price AL, Neale BM. LD Score regression distinguishes confounding from polygenicity in genome-wide association studies. *Nat Genet*. Mar 2015;47:291-295.

47. https://uk.mathworks.com/products/matlab.html.

48. Orini M, Graham AJ, Martinez-Naharro A, Andrews CM, de Marvao A, Statton B, Cook SA, O'Regan DP, Hawkins PN, Rudy Y, et al. Noninvasive Mapping of the Electrophysiological Substrate in Cardiac Amyloidosis and Its Relationship to Structural Abnormalities. *J Am Heart Assoc*. 2019;8:e012097.

49. Manriquez AI, Zhang Q. An algorithm for QRS onset and offset detection in single lead electrocardiogram records. *Conf Proc IEEE Eng Med Biol Soc*. 2007;2007:541-544.

50. Bihlmeyer NA, Brody JA, Smith AV, et al. ExomeChip-Wide Analysis of 95 626 Individuals Identifies 10 Novel Loci Associated With QT and JT Intervals. *Circ Genom Precis Med*. 01 2018;11:e001758. doi:10.1161/CIRCGEN.117.001758

51. van Setten J, Verweij N, Mbarek H, et al. Genome-wide association meta-analysis of 30,000 samples identifies seven novel loci for quantitative ECG traits. *Eur J Hum Genet*. 06 2019;27:952-962.

52. Willer CJ, Li Y, Abecasis GR. METAL: fast and efficient meta-analysis of genomewide association scans. *Bioinformatics*. 2010;26:2190-2191.

53. Purcell S, Neale B, Todd-Brown K, Thomas L, Ferreira MA, Bender D, Maller J, Sklar P, de Bakker PI, Daly MJ, Sham PC. PLINK: a tool set for whole-genome association and population-based linkage analyses. *Am J Hum Genet*. 2007;81:559-575.

54. Bowden J, Del Greco M F, Minelli C, Davey Smith G, Sheehan N, Thompson J. A framework for the investigation of pleiotropy in two-sample summary data Mendelian randomization. *Stat Med*. 2017;36:1783-1802.

55. Bowden J, Davey Smith G, Burgess S. Mendelian randomization with invalid instruments: effect estimation and bias detection through Egger regression. *Int J Epidemiol*. 2015;44:512-525.

American Heart Association.

Circulation: Genomic and Precision Medicine

This article is published in its accepted form; it has not been copyedited and has not appeared in an issue of the journal. Preparation for inclusion in an issue of *Circulation: Genomic and Precision Medicine* involves copyediting, typesetting, proofreading, and author review, which may lead to differences between this accepted version of the manuscript and the final published version.

DOI: 10.1161/CIRCGEN.120.003231

56. Bowden J, Davey Smith G, Haycock PC, Burgess S. Consistent Estimation in Mendelian Randomization with Some Invalid Instruments Using a Weighted Median Estimator. *Genet Epidemiol*. 2016;40:304-314.

57. Verbanck M, Chen CY, Neale B, Do R. Detection of widespread horizontal pleiotropy in causal relationships inferred from Mendelian randomization between complex traits and diseases. *Nat Genet*. 2018;50:693-698.

American Heart Association.

Circulation: Genomic and Precision Medicine

This article is published in its accepted form; it has not been copyedited and has not appeared in an issue of the journal. Preparation for inclusion in an issue of *Circulation: Genomic and Precision Medicine* involves copyediting, typesetting, proofreading, and author review, which may lead to differences between this accepted version of the manuscript and the final published version.

Table 1: Study characteristics for each ECG cohort and combined

	Calcium GWAS cohort	ECG cohorts		
		IMAGING-UKB	EST-UKB	Combined
**No. of individuals**	305,349	29,683	46,543	76,226
**Sex (Female)**	53.0%	52.7%	53.3%	53.1%
**Age (years)**	58 (50 – 63)	64 (58 - 69)	59 (51 - 64)	61 (54 - 66)
**BMI**	26.7 (24.1 – 29.9)	25.3 (22.9 - 28.1)	26.4 (24.0 - 29.4)	26.0 (23.5 - 28.9)
**Height (cm)**	168 (162 – 176)	170 (163 - 177)	169 (162 - 176)	169 (163 - 176)
**Systolic BP (mmHg)**	136.5 (124.5 – 149.5)	133.5 (122.5 – 146.0)	135.5 (124.0 – 148.0)	135.0 (123.5 – 147.5)
**Diastolic BP (mmHg)**	82.0 (75.0 – 89.0)	81.0 (74.5 – 88.0)	81.5 (75.0 – 88.0)	81.5 (75.0 – 88.0)
**Pulse rate (bpm)**	68.5 (61.5 – 76)	67 (60.5 – 74.0)	67 (61 – 74)	67 (60.5 – 74.0)
**Calcium (mmol/L)**	2.37 (2.32 – 2.43)	2.37 (2.32 – 2.43)	2.39 (2.33 – 2.45)	2.38 (2.32 – 2.44)
**Corrected calcium (mmol/L)**	2.27 (2.22 – 2.32)	2.26 (2.22 – 2.31)	2.27 (2.22 – 2.32)	2.27 (2.22 – 2.32)
***RR interval (ms)**	-	985 (885 - 1091)	852 (764 - 947)	900 (799 - 1013)
***QT (ms)**	-	398 (378 - 419)	356.5 (340 - 374)	370.5 (349 - 396)
***JT (ms)**	-	313 (294 - 335)	270 (253.5 - 287)	284 (263 - 311)
***QRS (ms)**	-	84 (77 - 91)	86 (83 - 91)	85 (81 - 91)

Continuous variables are reported as median (interquartile range). No.: Number, BMI: body mass index, cm: centimetres, mmHg: millimetre of mercury, bpm: beats per minute, mmol/L: millimole per litre, ms: milliseconds. *indicates measures derived from ECG analysis

Circulation: Genomic and Precision Medicine

American Heart Association.

This article is published in its accepted form; it has not been copyedited and has not appeared in an issue of the journal. Preparation for inclusion in an issue of *Circulation: Genomic and Precision Medicine* involves copyediting, typesetting, proofreading, and author review, which may lead to differences between this accepted version of the manuscript and the final published version.

DOI: 10.1161/CIRCGEN.120.003231

Table 2: Association between serum total calcium concentration and measures of ventricular depolarisation and repolarisation using Mendelian Randomization

		Inverse-median weighted		Median-Weighted		MR-Egger		MR-PRESSO outlier adjusted	
	No. SNPs	Beta (95% CI)	P-value	Beta (95% CI)	P-value	Beta (95% CI)	P-value	Beta (95% CI)	P-value
**QT interval**	205	-3.01 (-3.99 - -2.03)	$1.10x10^{-9}$	-4.02 (-5.04 - -3.00)	$1.59x10^{-14}$	-4.61 (-6.43 - -2.79)	$1.52x10^{-6}$	-3.24 (-3.98 - -2.50)	$1.23x10^{-14}$
**JT interval**	205	-2.89 (-3.87 - -1.91)	$4.36x10^{-9}$	-4.24 (-5.28 - -3.20)	$9.40x10^{-16}$	-4.51 (-6.31 - -2.71)	$2.09x10^{-6}$	-3.2 (-4.00 - -2.40)	$3.69x10^{-13}$
**QRS duration**	205	-0.2 (-0.49 - 0.10)	0.18	-0.44 (-0.93 – 0.05)	0.08	-0.13 (-0.70 – 0.44)	0.65	-0.14 (-0.41 – 0.13)	0.32

Beta = effect size (ms change per 0.1mmol/L).

American Heart Association.
Circulation: Genomic and Precision Medicine

Figure Legends:

Figure 1. Workflow indicating the methods for GWAS of serum calcium and ECG traits.
MAF: minor allele frequency, INFO: Imputation quality score, SNP: Single nucleotide polymorphism, Array: Indicator for UK Biobank (UKBB) or UK BiLEVE (UKBL) array to adjust for genotyping chip, GWAS: Genome wide association study, GWS: Genome wide significant ($P < 5x10^{-8}$), MR: Mendelian Randomization
*Phenotypic exclusions included a prior diagnosis of myocardial infarction or heart failure, QRS duration > 120ms or RBBB/LBBB on ECG, pacemaker in-situ, currently pregnant, or taking digitalis medication, class I / III anti-arrhythmics or specific QT prolongation medication
†Indicators of poor genotype quality included high heterozygosity / missingness / sex mis-match

Circulation: Genomic and Precision Medicine

Figure 2A. Scatter plot for mendelian randomisation serum total calcium-QT analyses.
Scatter plot of individual variant regression coefficients with Inverse-variance weighted, Weighted-median, and MR Egger slope estimates. B**.** Scatter plot for mendelian randomisation serum total calcium-JT analyses. Scatter plot of individual variant regression coefficients with Inverse-variance weighted, Weighted-median, and MR Egger slope estimates. **C.** Scatter plot for mendelian randomisation serum total calcium-QRS analyses.
Scatter plot of individual variant regression coefficients with Inverse-variance weighted, Weighted-median, and MR Egger slope estimates.

This article is published in its accepted form; it has not been copyedited and has not appeared in an issue of the journal. Preparation for inclusion in an issue of *Circulation: Genomic and Precision Medicine* involves copyediting, typesetting, proofreading, and author review, which may lead to differences between this accepted version of the manuscript and the final published version.

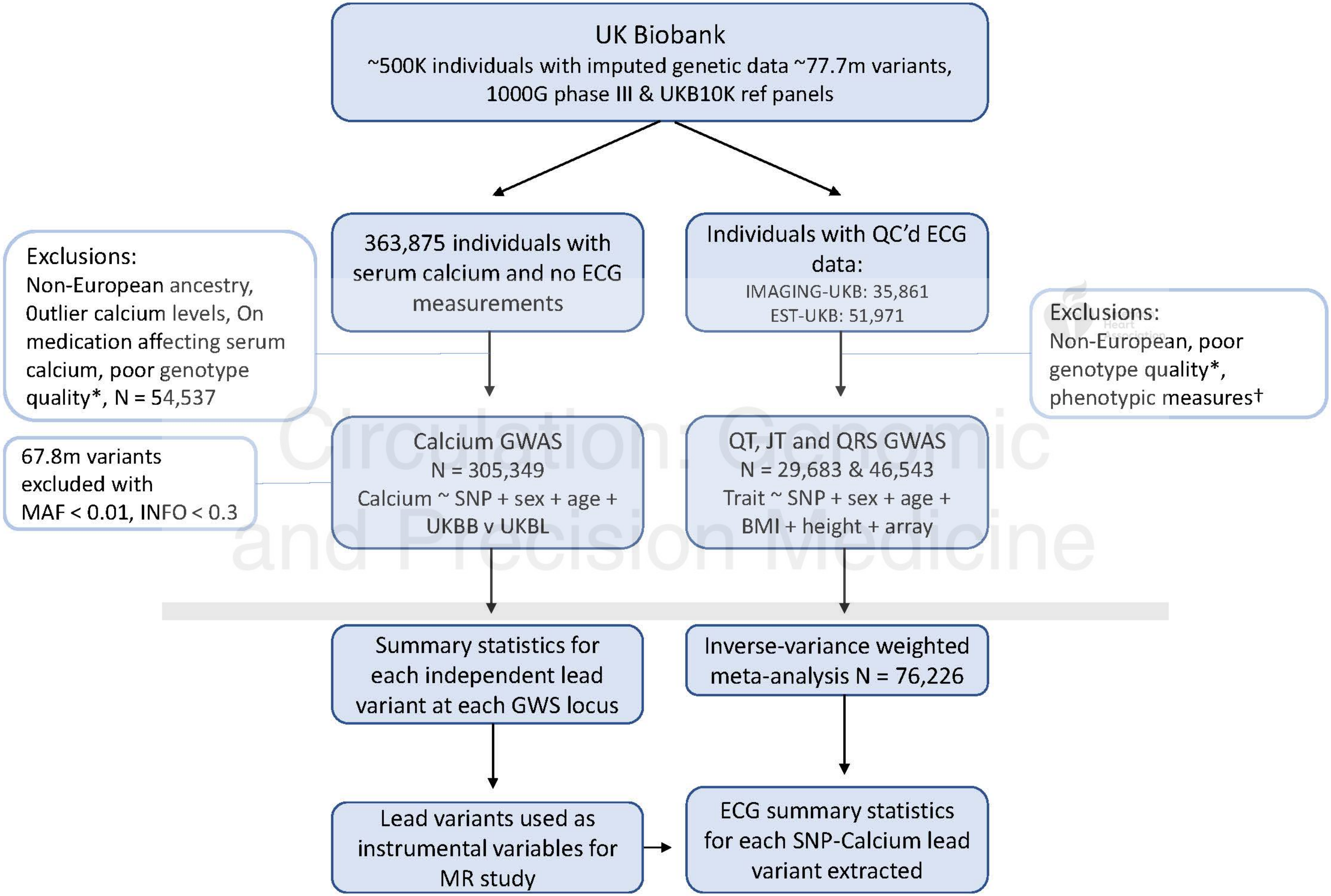

Circulation: Genomic and Precision Medicine

A

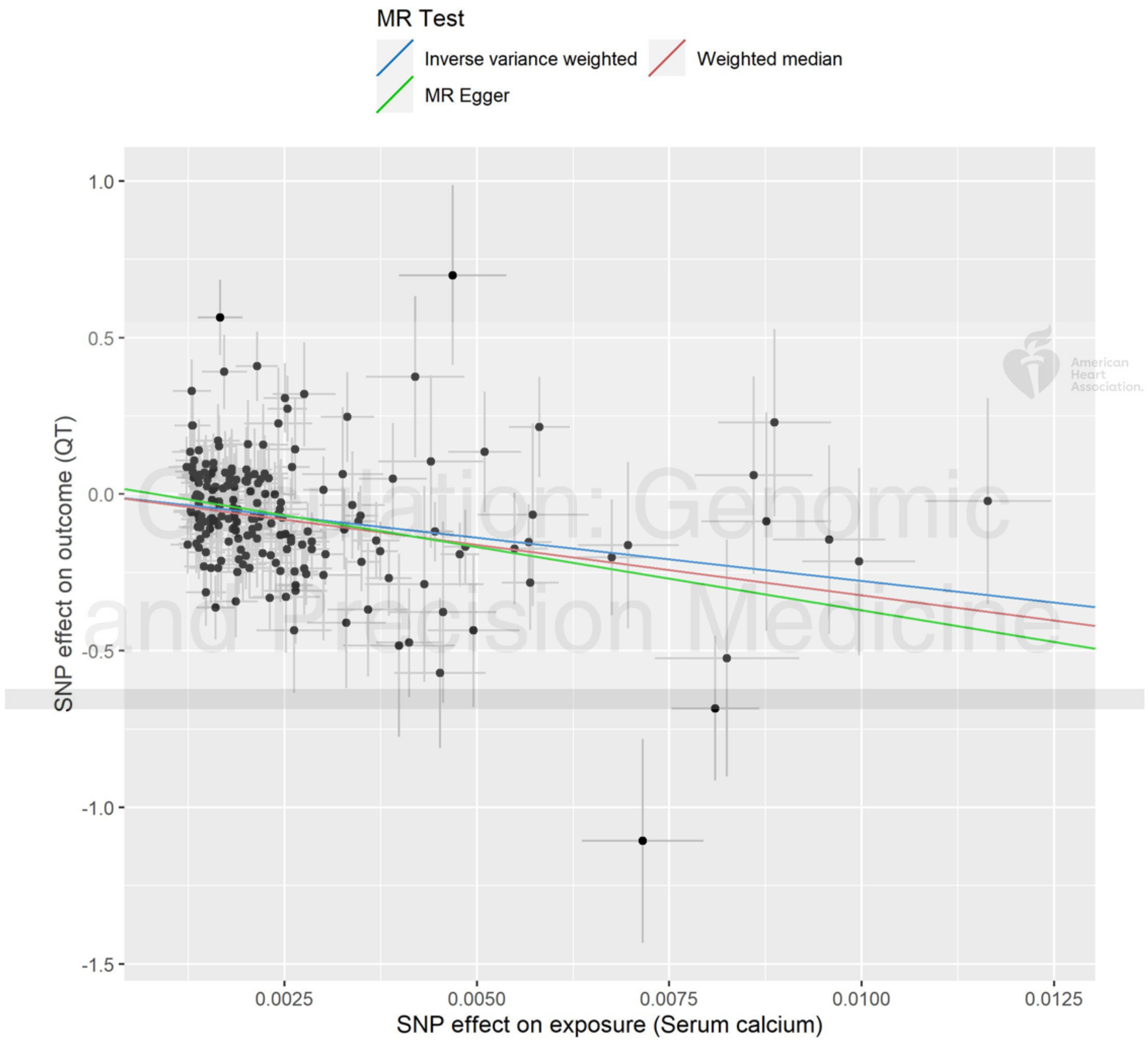

MR Test
Inverse variance weighted
Weighted median
MR Egger
1.0
0.5
0.0
-0.5
-1.0
-1.5
0.0025
0.0050
0.0075
0.0100
0.0125
SNP effect on outcome (QT)
SNP effect on exposure (Serum calcium)
American Heart Association.
Circulation: Genomic and Precision Medicine

B

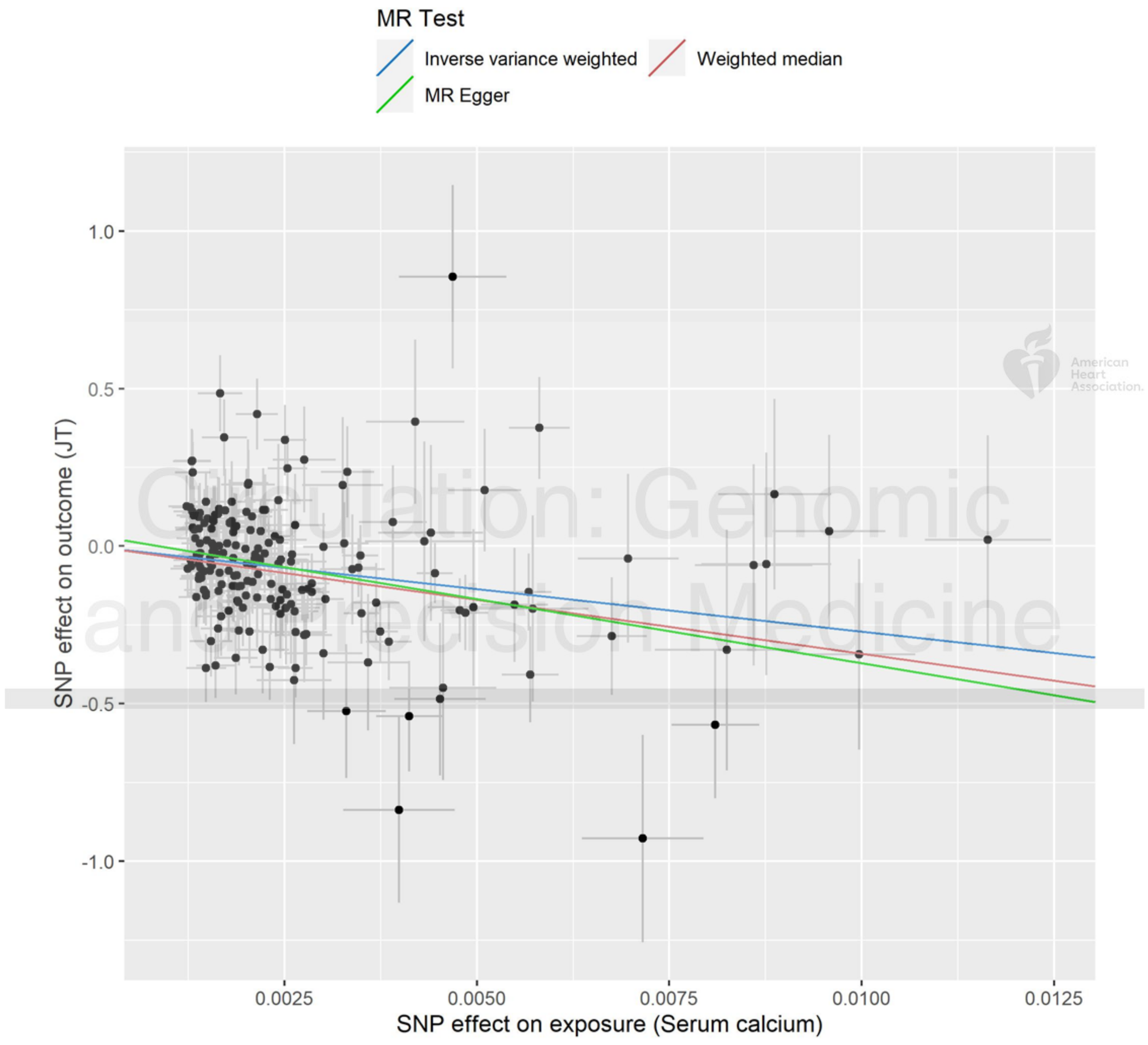

MR Test
Inverse variance weighted
Weighted median
MR Egger
1.0
0.5
0.0
-0.5
-1.0
SNP effect on outcome (JT)
0.0025
0.0050
0.0075
0.0100
0.0125
SNP effect on exposure (Serum calcium)
American Heart Association.
Circulation: Genomic and Precision Medicine

C

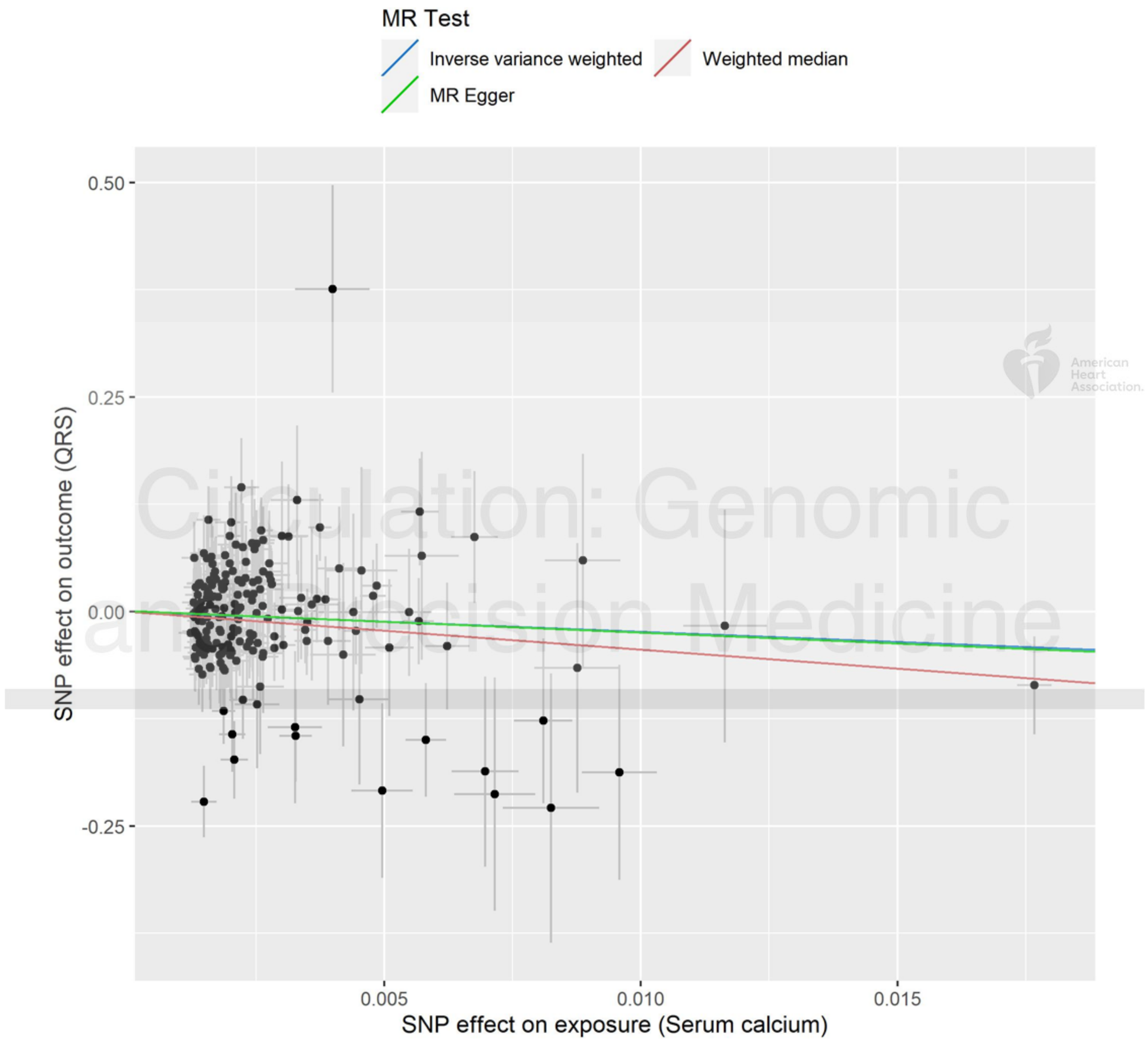
MR Test
Inverse variance weighted
Weighted median
MR Egger
0.50
0.25
0.00
-0.25
0.005
0.010
0.015
SNP effect on outcome (QRS)
SNP effect on exposure (Serum calcium)
American Heart Association.
Circulation: Genomic and Precision Medicine
